# Pregnancy outcomes after first-trimester treatment with artemisinin derivatives versus non-artemisinin antimalarials: a systematic review and individual patient data meta-analysis

**DOI:** 10.1016/S0140-6736(22)01881-5

**Published:** 2023-01-14

**Authors:** Makoto Saito, Rose McGready, Halidou Tinto, Toussaint Rouamba, Dominic Mosha, Stephen Rulisa, Simon Kariuki, Meghna Desai, Christine Manyando, Eric M Njunju, Esperanca Sevene, Anifa Vala, Orvalho Augusto, Christine Clerk, Edwin Were, Sigilbert Mrema, William Kisinza, Josaphat Byamugisha, Mike Kagawa, Jan Singlovic, Mackensie Yore, Anna Maria van Eijk, Ushma Mehta, Andy Stergachis, Jenny Hill, Kasia Stepniewska, Melba Gomes, Philippe J Guérin, Francois Nosten, Feiko O ter Kuile, Stephanie Dellicour

**Affiliations:** a WorldWide Antimalarial Resistance Network, Oxford, UK; b Infectious Diseases Data Observatory, Oxford, UK; c Centre for Tropical Medicine and Global Health, Nuffield Department of Medicine, University of Oxford, Oxford, UK; d Division of Infectious Diseases, Advanced Clinical Research Center, Institute of Medical Science, University of Tokyo, Tokyo, Japan; e Shoklo Malaria Research Unit, Faculty of Tropical Medicine, Mahidol University, Mae Sot, Thailand; f Clinical Research Unit of Nanoro, Institut de Recherche en Sciences de la Santé, Nanoro, Burkina Faso; g Ifakara Health Institute, Rufiji, Tanzania; h School of Medicine and Pharmacy, University Teaching Hospital of Kigali, University of Rwanda, Kigali, Rwanda; i Kenya Medical Research Institute, Centre for Global Health Research, Kisumu, Kenya; j Centers for Disease Control and Prevention, Atlanta, GA, USA; k Tropical Diseases Research Centre, Ndola, Zambia; l Department of Basic Sciences, Copperbelt University, Ndola, Zambia; m Faculty of Medicine, Eduardo Mondlane University, Maputo, Mozambique; n Centro de Investigação em Saúde de Manhiça, Manhiça, Mozambique; o School of Public Health, University of Ghana, Dodowa, Ghana; p Department of Reproductive Health, Moi University, Eldoret, Kenya; q National Institute of Medical Research, Amani Medical Research Centre, Muheza, Tanzania; r Department of Obstetrics and Gynaecology, Makerere University, Kampala, Uganda; s Icon, Prague, Czech Republic; t VA Los Angeles and University of California, Los Angeles National Clinician Scholars Program, VA Greater Los Angeles Healthcare System Health Services Research and Development Service Center of Innovation, Los Angeles, CA, USA; u Department of Clinical Sciences, Liverpool School of Tropical Medicine, Liverpool, UK; v Centre for Infectious Disease Epidemiology and Research, University of Cape Town, Cape Town, South Africa; w Department of Pharmacy, School of Pharmacy, and Department of Global Health, School of Public Health, University of Washington, Seattle, WA, USA; x UNICEF/UNDP/World Bank/WHO Special Programme for Research and Training in Tropical Diseases, Geneva, Switzerland; y School of Public Health and Community Medicine, Institute of Medicine, University of Gothenburg, Gothenburg, Sweden

## Abstract

**Background:**

Malaria in the first trimester of pregnancy is associated with adverse pregnancy outcomes. Artemisinin-based combination therapies (ACTs) are a highly effective, first-line treatment for uncomplicated *Plasmodium falciparum* malaria, except in the first trimester of pregnancy, when quinine with clindamycin is recommended due to concerns about the potential embryotoxicity of artemisinins. We compared adverse pregnancy outcomes after artemisinin-based treatment (ABT) versus non-ABTs in the first trimester of pregnancy.

**Methods:**

For this systematic review and individual patient data (IPD) meta-analysis, we searched MEDLINE, Embase, and the Malaria in Pregnancy Library for prospective cohort studies published between Nov 1, 2015, and Dec 21, 2021, containing data on outcomes of pregnancies exposed to ABT and non-ABT in the first trimester. The results of this search were added to those of a previous systematic review that included publications published up until November, 2015. We included pregnancies enrolled before the pregnancy outcome was known. We excluded pregnancies with missing estimated gestational age or exposure information, multiple gestation pregnancies, and if the fetus was confirmed to be unviable before antimalarial treatment. The primary endpoint was adverse pregnancy outcome, defined as a composite of either miscarriage, stillbirth, or major congenital anomalies. A one-stage IPD meta-analysis was done by use of shared-frailty Cox models. This study is registered with PROSPERO, number CRD42015032371.

**Findings:**

We identified seven eligible studies that included 12 cohorts. All 12 cohorts contributed IPD, including 34 178 pregnancies, 737 with confirmed first-trimester exposure to ABTs and 1076 with confirmed first-trimester exposure to non-ABTs. Adverse pregnancy outcomes occurred in 42 (5·7%) of 736 ABT-exposed pregnancies compared with 96 (8·9%) of 1074 non-ABT-exposed pregnancies in the first trimester (adjusted hazard ratio [aHR] 0·71, 95% CI 0·49–1·03). Similar results were seen for the individual components of miscarriage (aHR=0·74, 0·47–1·17), stillbirth (aHR=0·71, 0·32–1·57), and major congenital anomalies (aHR=0·60, 0·13–2·87). The risk of adverse pregnancy outcomes was lower with artemether–lumefantrine than with oral quinine in the first trimester of pregnancy (25 [4·8%] of 524 *vs* 84 [9·2%] of 915; aHR 0·58, 0·36–0·92).

**Interpretation:**

We found no evidence of embryotoxicity or teratogenicity based on the risk of miscarriage, stillbirth, or major congenital anomalies associated with ABT during the first trimester of pregnancy. Given that treatment with artemether–lumefantrine was associated with fewer adverse pregnancy outcomes than quinine, and because of the known superior tolerability and antimalarial effectiveness of ACTs, artemether–lumefantrine should be considered the preferred treatment for uncomplicated *P falciparum* malaria in the first trimester. If artemether–lumefantrine is unavailable, other ACTs (except artesunate–sulfadoxine–pyrimethamine) should be preferred to quinine. Continued active pharmacovigilance is warranted.

**Funding:**

Medicines for Malaria Venture, WHO, and the Worldwide Antimalarial Resistance Network funded by the Bill & Melinda Gates Foundation.

## Introduction

*Plasmodium falciparum* infection in early pregnancy impairs placental vasculogenesis and angiogenesis,^1^, ^2^, ^3^, ^4^ and is associated with gestational hypertension, maternal anaemia, pregnancy loss, preterm birth, intrauterine growth restriction, and low birthweight.^2^, ^4^, ^5^, ^6^, ^7^, ^8^, ^9^, ^10^ In areas with stable malaria transmission, more than 60% of malaria infections during pregnancy are estimated to occur in the first trimester.^11^, ^12^, ^13^, ^14^ During this period, most pregnancies are not protected by insecticide-treated nets, which are provided at the first antenatal care visit that typically happens in the second or third trimester,^15^ or by intermittent preventive treatment in pregnancy (IPTp) with the antifolate sulfadoxine–pyrimethamine, which is contraindicated in the first trimester. Therefore, effective recognition and prompt, safe, and effective treatment of malaria is a priority in the first trimester of pregnancy to protect the parent and the fetus.

**Research in context**

**Evidence before this study**

Until late 2022, WHO treatment guidelines recommend 7 days of quinine (with clindamycin, if available) for treating uncomplicated *Plasmodium falciparum* malaria in the first trimester of pregnancy, despite its poor tolerability, adherence, and effectiveness. In 2017, a systematic literature review and meta-analysis assessed the safety of artemisinin derivatives in the first trimester of human pregnancies, showing no difference in the risk of miscarriage, stillbirth, and congenital anomalies between artemisinin-exposed and quinine-exposed pregnancies. In 2021, WHO invited us to update the evidence on the safety of antimalarials in the first trimester of pregnancy to establish whether artemisinin-based combination therapy (ACT) could be reconsidered for the treatment of malaria in the first trimester. No relevant meta-analyses on the clinical safety of artemisinin derivatives in the first trimester were identified through our literature search, except the one published in 2017.

**Added value of this study**

This Article, by use of a one-stage, individual patient data (IPD), meta-analysis approach, presents current information on the safety of artemisinin compounds in the first trimester of pregnancy. This analysis includes data from two additional studies (five cohorts), one updated dataset from a previously included study, and the four studies included in the previous systematic review in 2017. Compared with the previous meta-analysis, in which only aggregated data were available from the largest contributing site and sites with no events did not contribute to the meta-analysis, this update includes IPD from all eligible studies and analysed all eligible data. We show that the risks of adverse pregnancy outcomes in pregnancies exposed to artemisinin-based treatments (ABTs) and non-ABTs in the first trimester were similar (adjusted hazard ratio [aHR] 0·71, 95% CI 0·49–1·03). Similar results were seen for the individual components of miscarriage (aHR=0·74, 0·47–1·17), stillbirth (aHR=0·71, 0·32–1·57), and major congenital anomalies (aHR=0·60, 0·13–2·87). First-trimester treatment with ACTs was associated with a lower risk of adverse pregnancy outcomes (0·59, 0·39–0·89) than oral non-ABTs. Similarly, artemether–lumefantrine was associated with a lower risk of adverse pregnancy outcomes than the standard of care with oral quinine (0·58, 0·36–0·92).

**Implications of all the available evidence**

Malaria in the first trimester can have severe consequences for the pregnancy. The currently available safety data (ie, no evidence of embryotoxicity and teratogenicity associated with first-trimester malaria treatment), together with the superior tolerability, higher efficacy, longer duration of post-treatment prophylaxis, and wide availability of ACTs, suggest that artemether–lumefantrine, with the most safety evidence available, should replace quinine-based regimens as the preferred treatment for uncomplicated *P falciparum* malaria in the first trimester of pregnancy. Other ACTs, except those with a first-trimester contraindicated partner drug (eg, artesunate–sulfadoxine–pyrimethamine), should be considered if artemether–lumefantrine is unavailable. Implementing new strategies to ensure the creation of robust evidence on the benefit–risk profile of antimalarials for treatment in the first trimester of pregnancy will be crucial in ensuring that individuals at hig risk (ie, people who are pregnant in the first trimester) can access the best treatments.

Until late 2022, WHO treatment guidelines for uncomplicated *P falciparum* malaria in the first trimester recommend a 7-day course with quinine combined with clindamycin (if available),^16^ which is poorly tolerated, poorly adhered to, and less effective than artemisinin-based combination therapies (ACTs).^17^, ^18^ ACTs have been the recommended first-line treatment in the second and third trimesters of pregnancy and in all other patient groups since 2006.^16^ However, ACTs have not been recommended in the first trimester of pregnancy due to concerns about the potential embryotoxicity and teratogenicity of the artemisinin class of compounds reported in animal studies.^19^, ^20^ The effects included embryo resorption, pregnancy loss, and congenital anomalies including skeletal defects (eg, shortened or bent long bones and scapulae, misshapen ribs, cleft sternebrae, and incompletely ossified pelvic bones) and heart defects (eg, ventricular septal and great vessel defects).^21^ The most sensitive period is assumed to be the 7 weeks between 6 and 12 weeks of gestation when primitive erythroblasts, the suggested primary target of embryotoxicity, predominantly circulate in human embryos.^22^

In 2017, WHO reviewed the evidence on the safety of artemisinin-based treatment (ABT) in the first trimester of pregnancy compared with non-artemisinin-based treatment (non-ABT).^23^, ^24^ This review, which included 30 618 pregnancies with 717 confirmed ABT first-trimester exposures from five studies, suggested no increase in the risk of miscarriage, stillbirth, or major congenital anomalies with ABTs compared with quinine in the first trimester. However, it did not change WHO treatment guidelines, which still recommend quinine-based regimens.^25^, ^26^ In 2021, WHO commissioned an update to the previous meta-analysis to assess whether ACTs should be reconsidered for the treatment of uncomplicated *P falciparum* malaria in the first trimester of pregnancy. This Article expands on the previous meta-analysis^23^ by including all studies published or completed since 2015 and by doing an individual patient data (IPD) meta-analysis.

## Methods

### Search strategy and acquisition of IPD

This systematic review and IPD meta-analysis was done according to a registed protocol (PROSPERO CRD42015032371) and is reported according to the PRISMA-IPD statement.^27^ The results of the previous systematic review, including studies published up to November, 2015, are reported elsewhere.^23^ We did an updated literature search for studies published between Nov 1, 2015, and Dec 21, 2021, by use of MEDLINE, Embase, and the Malaria in Pregnancy Library. The newly identified studies were combined with those identified in the previous literature search, which included studies published before 2015. A full list of search terms used is provided in the appendix (p 2). In addition, malaria researchers were contacted for any other potential data sources, inlcuding unpublished studies.

Eligible studies included prospective cohorts enrolling participants before pregnancy outcomes were known; had documented data on ABT exposure in the first trimester; and included comparator groups (women exposed to non-ABT or women unexposed to any antimalarials). There were no language restrictions. The Malaria in Pregnancy Library includes grey literature (eg, conference abstracts and PhD theses). Retrospective studies, case series, case reports, and studies not reporting pregnancy outcomes were excluded. After preliminary screening of article titles, abstracts were assessed for eligibility by AMvE and SD. AMvE did the search and documentation of identified and selected studies, and conflicts were resolved through discussion between reviewers. Finally, investigators of the eligible studies were invited to provide fully anonymised IPD for the meta-analysis.

### Participant eligibility, definition of exposure, and comparison groups

Women enrolled in included studies during pregnancy (ie, before pregnancy outcome was known) were included in the analysis. Pregnancies were excluded if data on the estimated gestational age (EGA) were missing, the fetus was confirmed to be unviable at enrolment, or exposure information was incomplete. Multiple gestation pregnancies (eg, twins) were also excluded as they have a higher risk of adverse pregnancy outcomes than singleton pregnancies. Women were included on the basis of their exposure to antimalarials regardless of confirmation of malaria infection, malaria species, or disease severity. The baseline characteristics (ie, age, gravidity, parity, previous history of miscarriage or stillbirth, height, bodyweight, marital status, HIV status, smoking status, drinking habits, literacy, education, and IPTp doses received) and calendar year were extracted at the individual level. Country, and calendar year if year was not available at the individual level, was extracted at the study level.

Pregnancies were categorised as being exposed to ABT, exposed to non-ABT, or neither (ie, unexposed). Only exposures confirmed by at least one written medical record or multiple data sources were included. Pregnancies with unconfirmed exposures were excluded to avoid potential misclassification of exposure. Two exposure risk periods were defined: the entire first trimester (EGA from ≥2 weeks and 0 days to <14 weeks and 0 days) or the putative embryo-sensitive period (EGA from ≥6 weeks and 0 days to <13 weeks and 0 days). Ethics approval was not required for this analysis.

### Outcomes

The primary outcome was a composite of either miscarriage (spontaneous fetal loss before EGA 28 weeks), stillbirth (fetal loss at or after EGA 28 weeks),^28^ or major congenital anomalies. Secondary outcomes included miscarriage, stillbirth, fetal loss (ie, miscarriage or stillbirth), and major congenital anomalies. The rationale for the use of a composite primary outcome was to assess the overall embryotoxicity and fetal toxicity of the artemisinins shown in animals, which resulted in either pregnancy loss or major congenital anomalies among livebirths, and to account for the competing nature of these outcomes. Major congenital anomalies were defined as any structural anomaly deemed to be of surgical, medical, or cosmetic importance at birth, detected by surface examination of livebirths by trained birth attendants (appendix p 2). Congenital anomalies with a suspected genetic cause (identified with the International Classification of Diseases, 10th edition, code Q90–99) were excluded.

### Statistical analysis

The primary analyses compared the risk of adverse pregnancy outcomes in ABT-exposed women with that in non-ABT-exposed pregnancies (the reference group) for the two risk periods by using adjusted hazard ratio (aHR) with 95% CI. In addition, we compared the risks of pregnancies exposed to ABT or non-ABT with the risks in pregnancies unexposed to any antimalarials during the exposure risk period. We also analysed comparisons between ACTs (eg, oral ABT excluding artesunate monotherapy and artesunate–clindamycin) and oral non-ABTs, and between artemether–lumefantrine and oral quinine-based treatments. Crude prevalences of major congenital anomalies among livebirths are presented with 95% CIs by Wilson's method because of small percentages.

A one-stage, random-effects, IPD meta-analysis was done for each outcome based on the exposure status in the two different risk periods by use of Cox models with shared frailty to account for within-cohort clustering. The time to outcome was based on EGA (in weeks), with the observation time starting from the EGA at enrolment accounting for left truncation. The risk period was from an EGA of 2 weeks (or 28 weeks for stillbirth) until pregnancy outcome. The risk period in women who had not had an adverse pregnancy outcome was extended to 50 weeks (or 28 weeks for miscarriage) to distinguish women with missing outcomes who were censored at the last observation. The number of women included in the IPD meta-analysis is shown as the denominator regardless of the availability of the outcome, unless otherwise stated.

Exposure status was treated as a time-dependent variable. The time after enrolment but before exposure was treated as unexposed in the analysis. Women exposed to sulfadoxine–pyrimethamine in the first trimester of pregnancy, to both ABT and non-ABT during the risk period, or to unconfirmed antimalarials were censored at the time of these exposures. Pregnancies exposed to the same class of antimalarials more than once during the exposure risk period were included. Exposures outside each risk period (first trimester or embryo-sensitive period) were not considered. The Newcastle-Ottawa scale was used to assess the risk of bias.

Only three potential confounders identified a priori (age, gravidity, and calendar year) were available across all cohorts. We did sensitivity analyses using multiple imputations for missing potential confounders (appendix p 2). HIV status was missing in 75% (25 699 of 34 178) of women. Thus, a sensitivity analysis excluding confirmed women who were HIV-positive was done. E-values were used to quantify the effects of unmeasured confounders. These e-values represent the minimum risk ratio at which an unmeasured confounder needs to be associated with both the exposure and the outcome to make the lower limit of 95% CI exceed unity.^29^ Other sensitivity analyses were also done, excluding pregnancies with non-*P falciparum* malaria, excluding pregnancies with multiple exposures to the same class of antimalarials, and including unconfirmed exposures. An additional exploratory analysis was done to measure the effect of each exposure week on pregnancy outcomes (appendix p 2). The proportional hazard assumption for the exposure groups was tested by use of Schoenfeld residuals. For sensitivity analyses for handling clustering within cohorts, stratified and fixed-effects Cox models were fitted to the primary outcome. Statistical heterogeneity was not assessed quantitatively as not all cohorts had events in both ABT-exposed and non-ABT-exposed groups. However, analyses were repeated by removing one cohort at a time to assess whether there were any influential cohorts. Analyses were done with Stata 16.1 MP and R 4.1.2.

### Role of the funding source

The funders of the study had no role in study design, data collection, data analysis, data interpretation, or writing of the report.

## Results

**Figure 1:**
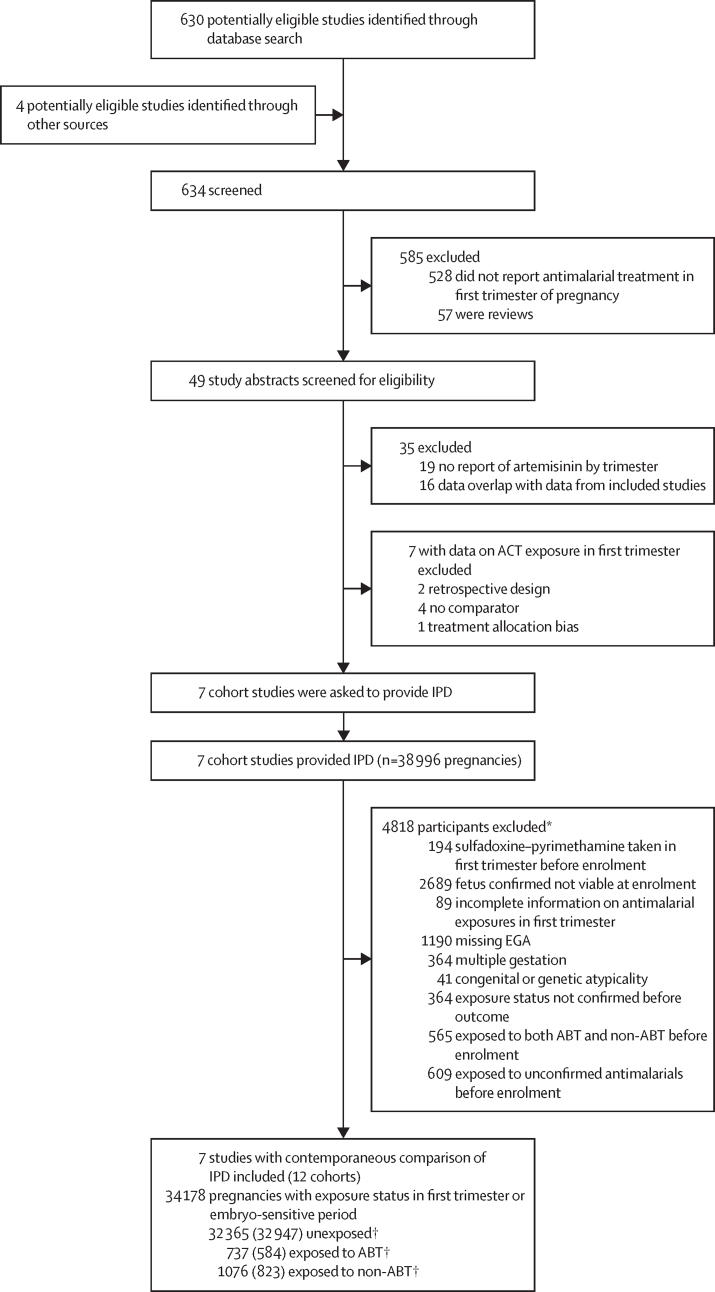
Study selection ABT=artemisinin-based treatment. ACT=artemisinin-based combination therapies. EGA=estimated gestational age. IPD=individual patient data. *Multiple reasons for exclusion allowed. †Data in parentheses are the number of women for whom data are available during the embryo-sensitive period.

The search identified 634 studies for screening, which included 371 additional studies since the previous review in 2017 (figure 1).^23^ Overall, 14 studies assessed ABT exposure in the first trimester of pregnancy. Seven of these studies were excluded because they did not meet the eligibility criteria (appendix pp 12–14). The remaining seven were included in our meta-analysis: four were also included in the previous publication,^30^, ^31^, ^32^, ^33^ one was an updated dataset from a previously published cohort,^34^, ^35^ one was an unpublished pregnancy cohort study in Burkina Faso,^36^ and one was an unpublished multicountry study with four cohorts from the WHO Special Programme for Research and Training in Tropical Diseases Pregnancy Exposure Registry.^37^ The study design and quality of the included and excluded studies are summarised in the appendix (pp 3–13). All studies were assessed as low risk of bias.

All seven eligible studies contributed IPD and involved 34 178 pregnancies (appendix p 15). They were done between 2000 and 2017 in nine countries: 11 cohorts in sub-Saharan Africa (n=12 268 pregnancies [5196 from Burkina Faso, 255 from Ghana, 1384 from Kenya, 734 from Mozambique, 1648 from Rwanda, 1939 from Tanzania, 177 from Uganda, and 935 from Zambia]) and one in Asia on the Thailand–Myanmar border (n=21 910). Compared with the previous review published in 2017,^23^ 8126 additional pregnancies and 60 additional artemisinin exposures were included in this meta-analysis that were not included in the previous publication, either because they are new data or due to differences in the eligibility criteria for statistical analyses.

The mean age was 26·1 years (SD 6·5), and 10 103 (29·7%) of 34 036 were primigravida (table 1). Pregnancy outcomes were available for 30 136 (88·2%) of all 34 178 pregnancies. Of the 34 178 pregnancies, 1813 (5·3%) had confirmed exposure to any antimalarial in the first trimester (1569 once, 200 twice, 40 three times, and four pregnancies were exposed four times), including 737 (2·2%) confirmed ABT-exposed pregnancies and 1076 (3·1%) confirmed non-ABT-exposed pregnancies. The 737 ABT-exposed pregnancies included 637 exposed to ACTs (of which 525 were exposed to artemether–lumefantrine; appendix pp 18–19). The 1076 non-ABT-exposed pregnancies included 917 exposed to oral quinine. The remaining 32 365 pregnancies were not exposed to any antimalarial in the first trimester. The analysis that was restricted to the embryo-sensitive period included 584 confirmed ABT-exposed pregnancies and 823 non-ABT-exposed pregnancies (appendix pp 16–17).

**Table 1:** Characteristics of women unexposed to antimalarials and women with confirmed exposure to ABT or non-ABT in the first trimester of pregnancy

		**Unexposed (n=32 365)**		ABT-exposed*(n=737)		**Non-ABT-exposed**†**(n=1076)**	
		n	Mean (SD) or n (%)	n	Mean (SD) or n (%)	n	Mean (SD) or n (%)
EGA at exposure, weeks		NA	NA	737	8·8 (3·0)	1076	8·9 (3·0)
Duration of follow-up, weeks		32 365	22·4 (9·9)	737	21·0 (9·3)	1076	23·1 (10·5)
Pregnancy outcome available		32 365	28 698 (88·7%)	737	669 (90·8%)	1076	769 (71·5%)
Age, years		32 268	26·1 (6·5)	737	25·6 (6·3)	1076	25·1 (6·6)
Gravidity		..	..	..	..	..	..
	1	32 226	9453 (29·3%)	736	314 (42·7%)	1074	336 (31·3%)
	2	32 226	6691 (20·8%)	736	128 (17·4%)	1074	214 (19·9%)
	≥3	32 226	16 082 (49·9%)	736	294 (39·9%)	1074	524 (48·8%)
Parity		..	..	..	..	..	..
	0	27 414	8175 (29·8%)	267	102 (38·2%)	999	354 (35·4%)
	1	27 414	6258 (22·8%)	267	60 (22·5%)	999	199 (19·9%)
	≥2	27 414	12 981 (47·4%)	267	105 (39·3%)	999	446 (44·6%)
Previous miscarriage		28 298	6078 (21·5%)	541	91 (16·8%)	1030	274 (26·6%)
Previous stillbirth		27 728	759 (2·7%)	535	10 (1·9%)	978	39 (4·0%)
Height (m)		1415	1·6 (0·1)	83	1·6 (0·1)	7	1·6 (0·1)
Bodyweight (kg)		1441	59·7 (9·5)	232	50·0 (9·5)	772	46·3 (6·7)
Married		24 767	23 978 (96·8%)	461	412 (89·4%)	907	898 (99·0%)
HIV positive		7777	643 (8·3%)	508	36 (7·1%)	194	5 (2·6%)
Current smoker		22 895	4805 (21·0%)	305	51 (16·7%)	827	305 (36·9%)
Any alcohol consumed during pregnancy		2058	295 (14·3%)	113	14 (12·4%)	28	9 (32·1%)
Literate		8467	5300 (62·6%)	65	30 (46·2%)	113	51 (45·1%)
Education		..	..	..	..	..	..
	No education	4635	907 (19·6%)	437	65 (14·9%)	102	6 (5·9%)
	Primary education	4635	2846 (61·4%)	437	255 (58·4%)	102	74 (72·5%)
	Secondary education or higher	4635	882 (19·0%)	437	117 (26·8%)	102	22 (21·6%)
IPTp doses		..	..	..	..	..	..
	0	6902	1504 (21·8%)	135	56 (41·5%)	183	39 (21·3%)
	1	6902	2202 (31·9%)	135	29 (21·5%)	183	60 (32·8%)
	2	6902	2692 (39·0%)	135	26 (19·3%)	183	74 (40·4%)
	3	6902	390 (5·7%)	135	18 (13·3%)	183	9 (4·9%)
	4	6902	114 (1·7%)	135	6 (4·4%)	183	1 (0·5%)
	NA	20 905	..	194	..	811	..
Gestational age measured by ultrasound		28 831	20 069 (69·6%)	497	248 (49·9%)	1000	621 (62·1%)
Location		..	..	..	..	..	..
	Burkina Faso	32 365	4980 (15·4%)	737	43 (5·8%)	1076	173 (16·1%)
	Ghana	32 365	246 (0·8%)	737	5 (0·7%)	1076	4 (0·4%)
	Kenya	32 365	1305 (4·0%)	737	74 (10·0%)	1076	5 (0·5%)
	Tanzania	32 365	1714 (5·3%)	737	156 (21·2%)	1076	69 (6·4%)
	Uganda	32 365	171 (0·5%)	737	3 (0·4%)	1076	3 (0·3%)
	Mozambique	32 365	710 (2·2%)	737	19 (2·6%)	1076	5 (0·5%)
	Rwanda	32 365	1571 (4·9%)	737	77 (10·4%)	1076	0
	Zambia	32 365	763 (2·4%)	737	166 (22·5%)	1076	6 (0·6%)
	Thailand–Myanmar border	32 365	20 905 (64·6%)	737	194 (26·3%)	1076	811 (75·4%)
Study year		..	..	..	..	..	..
	2000–04	32 365	5243 (16·2%)	737	55 (7·5%)	1076	374 (34·8%)
	2005–09	32 365	9986 (30·9%)	737	318 (43·1%)	1076	364 (33·8%)
	2010–17	32 365	17 136 (52·9%)	737	364 (49·4%)	1076	338 (31·4%)

Women are categorised according to the first exposure in the first trimester. Unexposed women represent pregnancies with no antimalarial exposure according to any sources during the first trimester. Pregnancies exposed to antimalarials in the first trimester contributed person-time to the unexposed group until they were exposed (with either confirmed or unconfirmed exposures). ABT=artemisinin-based treatment. EGA=estimated gestational age. IPTp=intermittent preventive treatment in pregnancy. n=number of women. NA=not applicable.

* Including 637 pregnancies exposed to artemisinin-based combination therapies (525 exposed to artemether–lumefantrine, 32 to artesunate–amodiaquine, 58 to artesunate–mefloquine, 19 to artenimol–piperaquine, and three to artesunate–atovaquone–proguanil), 95 to artesunate with or without clindamycin, and five to parenteral artesunate, on the basis of first exposure in the first trimester.

† Including 917 pregnancies exposed to oral quinine (715 exposed to quinine monotherapy and 202 to quinine plus clindamycin), nine to parenteral quinine, 147 to chloroquine, one to mefloquine, one to atovaquone–proguanil, and one to quinine plus mefloquine, on the basis of first exposure in the first trimester.

**Figure 2:**
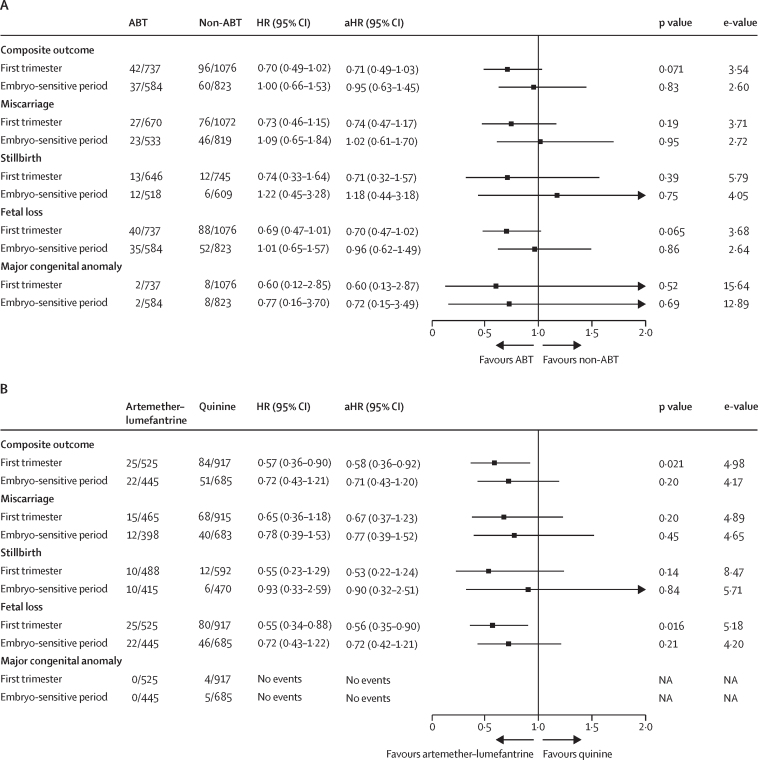
Adverse pregnancy outcomes in the first trimester and during the embryo-sensitive period in women exposed to antimalarials (A) Compares women treated with ABT with women treated with a non-ABT antimalarial. (B) Compares women treated with artemether–lumefantrine with women treated with an oral quinine-based treatment. The composite primary outcome includes miscarriage, stillbirth, or major congenital anomalies; fetal loss includes miscarriage or stillbirth. Adjusted by age group (<20 years, 20–29 years, 30–39 years, or ≥40 years), gravidity (1, 2, or ≥3 number of pregnancies, including the current pregnancy), and study year (2000–04, 2005–09, or 2010–17). A shared frailty Cox model was fitted to adjust for within-study clustering. The numbers in the ABT, non-ABT, artemether-lumefantrine, and quinine columns represent the pregnancies included in the unadjusted analysis. In the adjusted analysis, three women (one exposed to artemether–lumefantrine and two exposed to quinine) with a missing covariate (gravidity) were not included. ABT=artemisinin-based treatment. aHR=adjusted hazard ratio. HR=hazard ratio. NA=not available.

2531 (7·4%) of all 34 178 pregnancies had an adverse pregnancy outcome. The proportion of pregnancies with adverse outcomes in participants followed up until birth or other end of pregnancy was 2531 (8·4%) of 30 136. After excluding 165 women (one in the ABT group, two in the non-ABT group, and 162 in the unexposed group) whose covariate information was missing, 34 013 women contributed to the covariate-adjusted analysis. The aHR of adverse pregnancy outcomes in ABT-exposed pregnancies (42 [5·7%] of 736) compared with non-ABT-exposed pregnancies (96 [8·9%] of 1074) was 0·71 (95% CI 0·49–1·03) in the first trimester. The risk was similar between the exposure groups in the embryo-sensitive period (37 [6·3%] of 584 in the ABT group *vs* 60 [7·3%] of 822 in the non-ABT group; aHR 0·95, 0·63–1·45; figure 2A).

An analysis restricted to ACT versus oral non-ABT exposure in the first trimester showed that the risk of adverse pregnancy outcomes in pregnancies exposed to ACTs was lower than in oral non-ABT exposed pregnancies (aHR 0·59, 95% CI 0·39–0·89; appendix p 24). Most of these exposures were to artemether–lumefantrine and oral quinine. The risk of adverse pregnancy outcomes in pregnancies exposed to artemether–lumefantrine in the first trimester was lower than in pregnancies exposed to oral quinine in the first trimester (25 [4·8%] of 524 in the artemether–lumefantrine group *vs* 84 [9·2%] of 915 in the quinine group; aHR 0·58, 0·36–0·92) but not significantly different in the embryo-sensitive period (22 [4·9%] of 445 *vs* 51 [7·5%] of 684; aHR 0·71, 0·43–1·20; figure 2B). The numbers for the other ACTs were too small to do further analyses for specific ACTs.

The risk of adverse pregnancy outcomes did not differ between ABT-exposed pregnancies and unexposed pregnancies (aHR 0·92, 95% CI 0·67–1·26). By contrast, the risk was higher for non-ABT-exposed pregnancies than for unexposed pregnancies (aHR 1·30, 1·06–1·60; appendix p 20).

Further sensitivity analyses came to similar conclusions. The results of analyses restricted to pregnancies with only one exposure in the first trimester or pregnancies with *P falciparum* malaria were similar to those of the primary analysis (appendix pp 25–26). The results of multiple imputation models accounting for age group, gravidity, marital status, smoking status, previous history of miscarriage and stillbirth, and calendar year were similar to the primary model (appendix pp 28–29). All e-values exceeded 3·5 for the exposure analysis in the first trimester and exceeded 2·5 for the embryo-sensitive period, even after accounting for measured potential confounders.

In addition, a sensitivity analysis excluding pregnancies with known positive HIV status came to conclusions similar to the primary analyses (appendix p 31). A sensitivity analysis that included all unconfirmed exposures showed that the aHRs for ABT-exposed women compared with non-ABT-exposed women were similar to those obtained from the primary analysis that only included confirmed exposures (appendix pp 43–47). No single study changed the conclusion or the direction of the effect estimates when excluded from the analysis (appendix pp 39–42). The results were similar when only sub-Saharan African countries were included (appendix pp 39–42) and were not affected by different statistical models for handling clustering within cohorts (appendix p 30).

In an exploratory analysis assessing the effect by gestational week of exposure, there was no clear difference in aHRs in ABT-exposed pregnancies than in non-ABT-exposed pregnancies or unexposed pregnancies in a specific week (appendix p 27).

1910 miscarriages (6·0%) occurred among 32 042 pregnancies enrolled in or before the 28th week of gestation. The aHR of miscarriage in ABT-exposed pregnancies compared with non-ABT-exposed pregnancies in the first trimester was 0·74 (95% CI 0·47–1·17) and in the embryo-sensitive period was 1·02 (0·61–1·70; figure 2A). A similar pattern was observed for the risk in artemether–lumefantrine-exposed pregnancies compared with quinine-exposed pregnancies in the first trimester (0·67, 0·37–1·23) and in the embryo-sensitive period (0·77, 0·39–1·52; figure 2B). The risk of miscarriage was significantly higher in first-trimester pregnancies treated with non-ABT than in pregnancies not exposed to antimalarials in the first trimester, but this increased risk was not observed with ABTs (appendix p 20).

429 (1·5%) stillbirths occurred among 29 338 pregnancies followed up after 28 weeks of gestation. The aHR of stillbirths in ABT-exposed pregnancies compared with non-ABT-exposed pregnancies was 0·71 (95% CI 0·32–1·57) in the first trimester and 1·18 (0·44–3·18) in the embryo-sensitive period (figure 2A). A similar pattern was observed in artemether–lumefantrine-exposed pregnancies compared with quinine-exposed pregnancies in the first trimester (0·53, 0·22–1·24) and in the embryo-sensitive period (0·90, 0·32–2·51; figure 2B).

2339 (6·8%) fetal losses (miscarriages and stillbirths) occurred in 34 178 pregnancies. The aHR of fetal loss in ABT-exposed compared with non-ABT-exposed pregnancies was 0·70 (95% CI 0·47–1·02) in the first trimester and 0·96 (0·62–1·49) in the embryo-sensitive period (figure 2A). The risk was lower in artemether–lumefantrine-exposed pregnancies than in quinine-exposed pregnancies in the first trimester (0·56, 0·35–0·90), but was not significantly different in the embryo-sensitive period (0·72, 0·42–1·21; figure 2B).

192 (0·6%) major congenital anomalies were detected in 34 178 enrolled pregnancies, and 192 (0·7%, 95% CI 0·60–0·80) of 27 574 livebirths (table 2; appendix pp 21–22). Neither limb deformities or congenital heart defects, which were reported in animals, were observed in ABT-exposed pregnancies, although cardiac auscultation of neonates was systematically assessed only in one cohort^34^, ^35^ and other studies did not systematically screen for heart defects. The limb anomaly reported in the ABT-exposed group was a bilateral syndactyly, whereas rodent studies reported limb deformities, including bent or shortened long bones. The aHR of major congenital anomalies in ABT-exposed compared with non-ABT-exposed pregnancies was 0·60 (95% CI 0·13–2·87) in the first trimester and 0·72 (0·15–3·49) in the embryo-sensitive period (figure 2A). No major congenital anomalies were detected in the 482 livebirths from pregnancies exposed to artemether–lumefantrine in the first trimester (none [0%, 95% CI 0·00–0·79] of 482). The prevalence of major congenital anomalies in the quinine-exposed group was four (0·74%, 0·29–1·88) of 543.

**Table 2:** Summary of the distribution of major congenital anomalies by EUROCAT subgroups^38^

	**First trimester**			**Embryo-sensitive period**	
	ABT (n=623)	Non-ABT (n=681)	Unexposed (n=26 270)	ABT (n=503)	Non-ABT (n=558)
Any major congenital anomaly*	2 (0·32%)†	8 (1·17%)‡	182 (0·69%)	2 (0·40%)†	8 (1·43%)‡
Multiple congenital anomalies	0	1 (0·15%)	36 (0·14%)	0	1 (0·18%)
Nervous system	0	0	27 (0·10%)	0	0
Eye	0	0	8 (0·03%)	0	0
Ear, face, and neck	0	0	13 (0·05%)	0	0
Congenital heart defects§	0	1 (0·15%)	15 (0·07%)	0	1 (0·18%)
Orofacial clefts	1 (0·16%)	2 (0·29%)	30 (0·12%)	0	2 (0·36%)
Digestive system	0	0	20 (0·08%)	1 (0·20%)	0
Abdominal wall defects	0	0	10 (0·04%)	0	0
Urinary	0	0	4 (0·02%)	0	0
Genital	0	0	8 (0·03%)	0	0
Limb	1 (0·16%)	5 (0·73%)	46 (0·18%)	1 (0·20%)	5 (0·90%)
Other anomalies or syndromes	0	1 (0·15%)	24 (0·10%)	0	1 (0·18%)
Anomalies excluded from EUROCAT subgroups¶	0	0	3 (0·01%)	0	0

Data are n (%). ABT=artemisinin-based treatment.

* Some cases of congenital anomalies appear in multiple subgroups; therefore, the total in each column might not add up.

† The ABT in utero exposed cases of congenital anomalies were: (first trimester) one case of cleft lip and palate and one case of bilateral syndactyly; (embryo-sensitive period) one case of bilateral syndactyly and one case of imperforated anus. The case of imperforated anus was not counted in the first trimester analysis as the pregnancy was censored at 4 weeks of gestation due to exposure to chloroquine; because 4 weeks is not within the embryo-sensitive period, the pregnancy was not censored within this period and thus was included in the analysis of the embryo-sensitive period.

‡ The eight non-ABT in utero exposed cases of congenital anomalies (first trimester and embryo-sensitive period) were: two cases of cleft lip and palate; two cases of unilateral talipes; one case of syndactyly (both hands and feet) and bilateral talipes; one case of congenital heart defect; one case of amniotic banding on right hands, polydactyly, and one foot with missing toe; and one case of bilateral brachysyndactyly.

§ None of the studies were designed to systematically screen and detect congenital heart defects. 13 cases of congenital heart defects (12 unexposed and one non-ABT) were detected on the Thailand–Myanmar border, the only site systematically screening for heart murmurs. There were two cases of congenital heart defects detected in Kenya and one case detected in Tanzania (all unexposed). The cases of congenital heart defects included one fatal case at 5 months (non-ABT); three cases of heart murmur and other major anomalies; five of murmur and cyanosis; and seven confirmed diagnoses (marked cardiomegaly with increased pulmonary vasculature on chest x-ray, dysplastic pulmonary valve, suspected congenital rubella with heart murmur, tetralogy of Fallot, congenital atrioventricular block with heart murmur, pulmonary artery atresia, hypoplastic left heart syndrome, and an ectopia cardis).

¶ Two cases of inguinal hernia, and one case of fetal hydrops.

Compared with unexposed pregnancies, the risk of major congenital anomalies did not differ in both ABT-exposed pregnancies (aHR 0·99, 95% CI 0·24–4·03) and non-ABT pregnancies (1·65, 0·81–3·36; appendix p 20).

## Discussion

In this meta-analysis we found no evidence of embryotoxicity or teratogenicity based on the assessment of miscarriage, stillbirth, or major congenital anomalies associated with ABT exposure during the first trimester of pregnancy. Furthermore, the risk of the composite outcome in women exposed to ABTs in the first trimester was probably lower than in women exposed to non-ABT (aHR 0·71, 95% CI 0·49–1·03). This finding was robust as all sensitivity analyses showed results in the same direction and e-values were high. In analyses restricted to comparing ACTs with oral non-ABTs, ACTs were associated with a significantly lower risk of adverse pregnancy outcomes (aHR 0·59, 95% CI 0·39–0·89). Most of these ACTs were artemether–lumefantrine treatments, which were associated with a significantly lower risk of adverse pregnancy outcomes than pregnancies treated with oral quinine in the first trimester (aHR 0·58, 0·36–0·92).

The adverse effects of malaria in the first trimester of pregnancy need to be considered when interpreting antimalarial safety risks. Malaria in pregnancy is associated with a 33% increase in pregnancy loss,^39^ and this increase can be as high as 60% in the first trimester.^34^ Furthermore, malaria infection in the first trimester impairs placental villous and vascular development,^1^, ^2^, ^3^, ^4^ leading to fetal growth restriction, preterm birth, and pregnancy loss.^40^ This Article showed that the risk of miscarriage was significantly higher in first-trimester pregnancies treated with non-ABT than in pregnancies not exposed to antimalarials in the first trimester, which is expected because of the effect of malaria itself on adverse pregnancy outcomes. By contrast, an increased risk was not observed when ABTs were used to treat malaria in the first trimester of pregnancy. This finding suggests that prompt treatment with effective antimalarials can counteract some of the adverse effects of malaria infection in early pregnancy.

In animal models, including in rodents and monkeys, artemisinin (as a class) was reported to have embryotoxic effects. We did not observe any increased risks of miscarriage, fetal loss, or the composite adverse pregnancy outcome after ABT compared with non-ABT exposures in either the first trimester or embryo-sensitive period. Possible explanations for the observed differences between animals and humans have been proposed.^21^, ^41^ Infection with *P falciparum* might protect or reduce potential embryotoxicity in pregnant humans on ABT because artemisinins concentrate in infected red blood cells, which reduces the availability of free artemisinin and its derivatives.

Furthermore, the length and dose of exposure could be crucial. In some animal models (eg, rats), the nucleated primitive erythroblasts, which are the primary target of embryotoxicity, are produced in just a few days (about 3 days in rats, between days 10 and 13 after conception).^21^, ^22^, ^42^ If rats are treated with artemisinin when these primitive erythroblasts are predominant in the circulation, substantial depletion of primitive erythroblasts can occur, leading to fatal consequences for fetal development. In humans, however, primitive erythropoiesis occurs for 6 weeks, and transient reductions of erythroblasts by short-term exposure (if any) could thus be replenished by newly produced cells.^22^ In monkeys, toxicity was only observed when artesunate was administered at 12 mg/kg per day for 12 days or longer,^43^ suggesting that treatment courses shorter than 12 days are insufficient to cause substantial depletion of embryonic erythroblasts. Unlike in animal models, a short treatment course of 3–7 days with artesunate at a 2–4 mg/kg per day target dose in humans might not be sufficient to result in embryotoxicity or have a clinically significant effect. Our analysis showed no increased risk of embryotoxicity during EGA 6–12 weeks when primitive erythroblasts predominantly circulate in human embryos.^22^ Human exposure data provide the greatest degree of confidence on embryotoxicity,^44^ and this Article shows that although the results of animal toxicology studies help to identify potential teratogens, results need to be interpreted with caution as they might not always be directly applicable to humans.

In this Article, no major congenital anomalies were observed in the artemether–lumefantrine-exposed group, and the 95% CI estimates suggest that the prevalence of major congenital anomalies were between 0·00% and 0·79%. This upper confidence limit is similar to the 0·69% (95% CI 0·60–0·80) background rate of major congenital anomalies detected at birth by surface examination in the group unexposed to antimalarials and the rate of 0·74% (0·29–1·88) in the quinine-exposed group. The prevalence of major congenital anomalies at birth observed in our study was lower than the 2% reported in high-income countries.^45^ This lower observed prevalence partly reflects the exclusion of defects of suspected genetic cause and that the assessment was restricted to a surface examination just at birth. Only approximately 60% of anomalies are generally detected at birth.^46^ In the USA, the prevalence of major congenital anomalies detectable by surface examination within the first week after birth was approximately 1·3%.^47^ Furthermore, congenital heart defects, the most common major congenital anomalies with a prevalence of approximately 1% in the USA and Europe,^48^ were not adequately assessed in the included cohorts. Only one cohort (on the Thailand–Myanmar border)^34^, ^35^ used chest auscultation and assessment for severe cyanosis to screen for potential heart defects at birth. This site reported a prevalence of congenital heart defects of 0·1% (16 of 15 974) in the unexposed group, showing that additional detection methods like echocardiography might be needed, but paediatric echocardiography was not available in any of the included cohorts. Only congenital anomalies in liveborn babies were included in our analysis as autopsies were unavailable. Therefore, the composite adverse outcome, of which we did not observe any increased risk in ABT-exposed women, is a better measurement to assess the overall embryotoxicity and fetal toxicity than the individual components of the composite outcome and accounts for the competing nature of these outcomes. The high rate (ie, >50%) of embryolethal and teratogenic outcomes in rats, rabbits, and monkeys was not observed in the human data.^20^, ^21^

The benefits of 3-day ACTs for treating uncomplicated malaria in the first trimester of pregnancy compared with the 7-day, administered every 8 h courses of oral quinine include much better efficacy, tolerability, and adherence.^17^, ^18^, ^49^A 2020 systematic review of trials in the second and third trimester of pregnancy reported that malaria treatment failure with quinine was six times higher than with artemether–lumefantrine (aHR 6·11, 95% CI 2·57–14·54).^17^ The increased duration of post-treatment prophylaxis conferred by ACTs is another important benefit in pregnancy as they prevent new infections for several weeks, whereas quinine has no post-treatment prophylactic effect due to its short half-life.^50^, ^51^ Furthermore, adherence to quinine is low because it is associated with cinchonism, nausea, and hypoglycaemia.^17^, ^52^

Additionally, harmonising the first-line treatment of uncomplicated malaria in the first trimester with that in other trimesters and the rest of the population would simplify case-management practices, service delivery, and supply-chain management. As first-trimester pregnancy is the only indication for oral quinine, the supply of quinine and clindamycin is problematic in many countries; in some parts of sub-Saharan Africa, quinine is rarely available in public facilities and most first-trimester malaria is already treated with first-line ACTs.^53^, ^54^, ^55^, ^56^, ^57^

Although a cost-effectiveness analysis is beyond the scope of this paper, our results suggest that artemether–lumefantrine, and possibly other ACTs, are likely to be more cost-effective than quinine–clindamycin because of the disability-adjusted life-year associated with poorly treated malaria, the simple antimalarial supply management, and the case management of women of childbearing age that does not require screening for pregnancy before treatment.

This study has some limitations, partly due to the observational nature and scope of the existing data.^23^ Artemisinin safety data are available largely from observational studies because ACTs are not yet recommended in the first trimester by WHO. Although addressed in this meta-analysis, risks of bias, confounding, and heterogeneity (both clinical and methodological) are intrinsically higher in observational studies than in randomised trials. First, the range of potential confounding factors available across the datasets was small. However, the relatively high e-values (>3·5 in the first trimester and >2·5 in the embryo-sensitive period) are reassuring. This means that the minimum strength of the association an unmeasured confounder would need to have is a value of >2·5 for both the treatment and the outcome to conceal potential increased risks of adverse pregnancy outcomes. Confounders with e-values of more than 2 are uncommon in clinical research.^29^

Second, measurement error could have happened in specifying exposure status and estimating gestational age. Measurement error in specifying exposure status was why we only included confirmed exposures in the primary analysis. Gestational age might not be accurate and precise enough to assess the risks of adverse outcomes in short periods (eg, 1 week), although gestational age was estimated with ultrasound in 69% of pregnancies in this analysis.

Third, information on adherence and dosage was largely unavailable. Fourth, heterogeneity between studies was not quantified, although no specific cohorts unduly influenced the direction of the effect estimates. Fifth, we did not adjust for multiple comparisons. In addition to the composite primary outcome, we also presented the results of each individual component of the primary outcome and did several subgroup analyses.

Finally, except for artemether–lumefantrine, we could not assess the drug-specific effects of ACTs because of the few first-trimester antimalarial exposures to each ACT and the low incidence of outcomes. However, preclinical studies suggest that the safety concerns with artemisinins in early pregnancy are a class effect that would involve all artemisinin derivatives. The partner drugs of included ACTs are either considered likely to be safe (eg, lumefantrine or 4-aminoquinolines, including amodiaquine and piperaquine) or are already approved (eg, mefloquine) for use in the first trimester of pregnancy in some countries, such as the UK and the USA, although the possibility of synergistic toxicity might remain.^20^, ^58^ Sulfadoxine–pyrimethamine is an antifolate, and so the combination of artesunate–sulfadoxine–pyrimethamine is contraindicated in the first trimester of pregnancy. The safety data of pyronaridine in any trimester of pregnancy are scarce, although no safety signals were reported with pyronaridine in preclinical studies.^58^

A previous analysis from a single study done on the Thailand–Myanmar border, which was included in the current meta-analysis, implied a potential increased risk of adverse pregnancy outcomes with the combination of artesunate–mefloquine in the embryo-sensitive period.^34^ No further studies reported on this combination. The authors of that study suggested that this finding might reflect the fact that women treated with artesunate–mefloquine presented with fever in early pregnancy (EGA <10 weeks), when the risk of miscarriage is highest. Furthermore, fetal viability was not confirmed before treatment because artesunate–mefloquine was more commonly prescribed at outpatient clinics before individuals became aware of their pregnancy, or in the early 2000s when gestational ultrasound was not available in that cohort.^34^

A major strength of this Article was the contribution of IPD from all eligible studies and the updated methods, which could include all available data. As a result, the statistical power of the study was increased and we were able to standardise the definitions of exposure and outcomes and apply the same statistical models accounting for left truncation and the time-dependent nature of the exposure. To avoid recall bias and ensure appropriate comparisons, only studies with internal comparators and studies in which exposures were documented before the pregnancy outcome was known were eligible.

This Article highlights the challenges in obtaining quality data on the safety of antimalarials in the first trimester of pregnancy. Generating robust evidence on the benefits and risks of antimalarial drugs in the first trimester of pregnancy is time-consuming, resource-intensive, and challenging.^23^ Despite the large number of pregnancies exposed to ACTs in the first trimester, it has taken more than 20 years to accumulate more than 700 well documented pregnancies exposed to artemisinins in the first trimester. Pregnancy registries provide reliable data on the safety of specific medicines in pregnancy in the postmarketing phase, but these registries are time-consuming and resource-intensive. Therefore, complementary approaches, including active registries and interventional studies (ie, trials in the first trimester), should be considered to increase the creation of data needed for adequate benefit–risk assessment and to ensure the best antimalarials are available in a timely manner for the treatment of malaria in the first trimester of pregnancy.

In conclusion, our findings indicate a favourable risk–benefit profile for use of artemether–lumefantrine in the first trimester of pregnancy. This Article supports the recommendation by WHO to include artemether–lumefantrine as the preferred treatment option for uncomplicated *P falciparum* malaria in the first trimester of pregnancy.^59^ Other ACTs, except those with partner drugs contraindicated in the first trimester (eg, artesunate–sulfadoxine–pyrimethamine), are preferred to quinine if artemether–lumefantrine is unavailable. However, as with all drugs in pregnancy, a possible risk of rare adverse events or embryotoxicity cannot be excluded. Continued active pharmacovigilance should be encouraged and supported, particularly for ACTs other than artemether–lumefantrine and for new antimalarial drugs being developed.

## Data sharing

De-identified data are available from the Worldwide Antimalarial Resistance Network data repository (http://www.wwarn.org/working-together/sharing-data/accessing-data), which can be requested via the Worldwide Antimalarial Resistance Network Data Access Committee (https://www.wwarn.org/about-us/governance-people/data-access-committee).

## Declaration of interests

We declare no competing interests.
