# Supplementary material for: Pregnancy outcomes after first-trimester treatment with artemisinin derivatives versus non-artemisinin antimalarials: a systematic review and individual patient data meta-analysis
Source: Lancet. 2023 Jan 14;401(10371):118–30. doi: 10.1016/S0140-6736(22)01881-5 (PMC9874756; doi:10.1016/S0140-6736(22)01881-5)
Supplement: Supplementary appendix [file mmc1.pdf]

THE LANCET

Supplementary appendix

This appendix formed part of the original submission and has been peer reviewed. We post it as supplied by the authors.

Supplement to: Saito M, McGready R, Tinto H, et al. Pregnancy outcomes after first-trimester treatment with artemisinin derivatives versus non-artemisinin antimalarials: a systematic review and individual patient data meta-analysis. *Lancet* 2022; published online Nov 25. https://doi.org/10.1016/S0140-6736(22)01881-5.

Pregnancy outcomes after first-trimester treatment with artemisinin derivatives versus non-artemisinin antimalarials: A systematic review and individual patient data meta-analysis

Supplementary file

Contents

1. Supplementary Methods 2
Search terms 2
Congenital anomaly detection methods and inclusion 2
Multiple imputation methods for missing data 2
Exploratory analysis assessing exposures in each week 2
2. Study design and quality of the included and excluded studies 3
3. Baseline characteristics for analyses restricted to embryo-sensitive period analyses, and analyses comparing artemether-lumefantrine and oral quinine 16
4. Summary of primary analyses results using the unexposed women as the reference group 20
5. Description of major congenital anomalies by EUROCAT sub-groups 21
6. Subgroup analyses 23
6-1. Subgroup analyses by antimalarial type 23
6-2. Subgroup analyses restricted to single exposure during the exposure risk window 25
6-3. Subgroup analyses excluding non-falciparum malaria 26
6-4. Subgroup analyses by narrower time-band for the exposure risk window 27
7. Sensitivity analyses 28
7-1. Different approaches to handling missing data 28
7-2. Different approaches to handling within-cohort clustering 30
7-3. Analyses excluding confirmed HIV-positive women 31
7-4. Assessment of study-specific data and influence of individual cohorts 32
7-5. Analyses including confirmed and unconfirmed exposures 43
8. Supplemental references 48

1. Supplementary Methods

Search terms

The following terms were used with no restriction for language: (Pregnant women OR pregnan* AND malaria) AND (Artemisinin* OR "Artemisinin Combination Therapy" OR ACT OR artemether OR artesunate OR dihydroartemisinin OR treatment) AND (Pregnancy complication [mh] OR safety OR "serious adverse event" OR miscarriage OR stillbirth OR "pregnancy loss" OR "spontaneous abortion" OR "birth defect" OR congenital abnormalities OR "congenital malformations" OR "congenital anomalies") AND cohort study [mh] OR prospective [tw].

The updated search was limited to publications from 1 November 2015 to 21 December 2021.

Congenital anomaly detection methods and inclusion

Unlike the previous analysis,[1] the presence of multiple minor anomalies was not regarded as a major congenital anomaly in this analysis because of the high variability in detection and documentation of minor anomalies across sites. Postaxial polydactyly, type B was not considered a major congenital anomaly and was excluded from the analysis.

Chest auscultation was systematically conducted in one cohort (McGready, Thailand-Myanmar). Heart ultrasound was not available in any cohorts.

An International Birth Defects Assessment Panel established by WHO[2] reviewed all suspected cases of major congenital anomalies for three studies[2-4] (8 out of 12 cohorts) carried out in Africa. The Panel used standard criteria to determine which major anomalies could have been caused by teratogenic exposure and were eligible for inclusion in the analysis, and which resulted from genetic aetiology and to be excluded from the analysis.[2] The panel members were blinded to exposure status of the cases.

Multiple imputation methods for missing data

Multiple imputation was used for variables with less than 30% of missingness. Imputation was conducted using a joint modelling approach using *jomo* command in R (R Foundation for Statistical Computing, Vienna, Austria).[5] All variables assessed in the multivariable model (exposure group, age group(<20, 20s, 30s, >=40), gravidity group (1, 2, >=3), study year (2000–2004, 2005–2009, 2010–17), marital status (married or not), smoking status (smoker or not)), previous history of miscarriage (yes/no) and previous history of stillbirth (yes/no)) and parity group (0, 1, >=2) were included in the imputation model with a random intercept for each cohort. The variable for each component of the composite outcome, cumulative hazard (Nelson-Aalen estimator), entry time and exit time were also included as auxiliary variables in the imputation model. Imputation was conducted 25 times. The analysis results in each imputed dataset were then combined using Rubin's rules using the *mi estimate* command in Stata MP 16.1 (StataCorp, TX, USA).

Exploratory analysis assessing exposures in each week

An exploratory analysis was conducted to see any signals of increased risk in narrower exposure risk windows. For this analysis, four indicator variables were made for each gestational week (annotated here as week X): exposed to artemisinin-based treatment (ABT) on week X; exposed to non-ABT on week X; exposed to ABT on another week, and exposed to non-ABT on another week. The risk period started from week X, and women with only one exposure were included in this analysis. As the numbers of exposures and events each week were very small, the unexposed group was used as the reference group.

Additional information can be found at https://www.wwarn.org/working-together/study-groups/study-group-safety-artemisinin-based-therapies-treatment-malaria-first

2. Study design and quality of the included and excluded studies

Table 1. Characteristics of studies in the individual patient data meta-analysis

**Study Identifier**	Manyando, Zambia
**Investigator/Contact**	Christine Manyando
**Country**	Zambia
**Study Period**	October 2004 to July 2008
**Study Design**	Multi-centre, prospective cohort study
**Participants**	Pregnant women presenting for antenatal care (ANC) at four clinics
**Inclusion and Exclusion criteria**	Pregnant women were eligible for inclusion if they had received AL or SP for the treatment of malaria
**Exposure ascertainment**	Self-report through enrolment at ANC and exposure were verified by documentation from their outpatient clinic files
**Gestational age measurements**	Last menstrual period (LMP) date and ultrasound for a few cases or Dubowitz assessment if LMP was unavailable
**Follow up visits**	• Women visited the antenatal clinic for assessment of safety parameters at baseline/enrolment, four weeks post-enrolment, four weeks pre-delivery, at delivery, and at six weeks post-delivery.<br>• Infants were followed up at six weeks, 14 weeks, and at 12 months after birth.
**Outcome**	• The primary endpoint was the incidence of perinatal mortality (stillbirth or neonatal death within 7 days of birth).<br>• Secondary outcome measures were gestational age at delivery and birth weight adjusted for gestational age.<br>• Exploratory endpoints were assessed: frequency of spontaneous abortion, preterm delivery, neonatal mortality, maternal mortality, major and minor birth defects, and infant development
**Ethical review**	The study protocol was approved by the local Ethics Review Committee of the Tropical Diseases Research Centre, Zambia, and WHO Ethics Review Committee, Geneva. All participants or their parent/guardian (if the subject was a minor), gave written or finger-marked informed consent before study entry.
**Notes**	• Malaria diagnosis was clinically or parasitologically confirmed: malaria was unconfirmed in 82·0% of the AL and 87·2% of the SP exposure groups<br>• First trimester in the paper was defined as 2-12weeks post-LMP (exclusive).<br>• SP was the standard antimalarial treatment during pregnancy at the time of the study.<br>• Mean gestational age at enrolment was 24·6 weeks (SD 8·01) which is reflected by the low number of miscarriages detected (1% of pregnancies)<br>• 15 miscarriages reported in the manuscript represent 12 pregnancies (1 triplet and 1 twin pregnancy)<br>• Approximately 30% of women in the AL group and 38% in the SP exposure group were tested for HIV<br>• No information on marital status
**Bias assessment**	Overall low risk of bias (6/9 stars)<br>• Selection: 3 /4 stars - enrolled from 4 ANC clinics<br>• Comparability: 1 /2 stars - confounding by indication possible as not randomised/blinded allocation of treatment. SP comparator but not recommended in the first trimester and declining efficacy<br>• Outcome assessment: 3 /4 stars - the outcome was ascertained prospectively. Attrition was low: 4% (22/495) discontinued in AL exposure group and 6% (28/506) in SP exposure group before the end of pregnancy (14% and 18% by the end of study, respectively). Relatively late recruitment at ANC means only late miscarriages were captured.
**Citation**	Manyando C, Mkandawire R, Puma L, et al. Safety of artemether-lumefantrine in pregnant women with malaria: results of a prospective cohort study in Zambia. Malar J 2010; 9: 249.[6]

**Study Identifier**	Rulisa, Rwanda
**Investigator/Contact**	Stephen Rulisa
**Country**	Rwanda
**Study Period**	June 2007–July 2009
**Study Design**	Multi-centre (10 health facilities), prospective cohort study
**Participants**	Pregnant women presenting for treatment at selected health facilities
**Inclusion and Exclusion criteria**	Pregnant women above the age of 18 years were included in the study if the woman was to be treated with AL after diagnosis of uncomplicated *P. falciparum* malaria. A woman with a similar stage of pregnancy and without a history of previous or current treatment with AL in the existing pregnancy was selected at the same health centre during routine attendance at the antenatal clinic and invited to participate in the study as part of the control group.
**Exposure ascertainment**	• "prospective" ascertainment: women enrolled immediately after prescription of AL for malaria<br>• "Retrospective" ascertainment: women who, during antenatal clinic attendance, were found to have been treated with AL during that pregnancy if treatment could be verified from the patient prescription and treatment register at the health centre.<br>• unexposed group consisted of pregnant women with no history of previous or current treatment with AL in the existing pregnancy and without any signs or symptoms of malaria
**Gestational age measurements**	Last menstruation, fundal height and date of quickening were recorded and a correlation of at least two of the three factors was used for the gestational age determination. Gestational age was verified by ultrasound in a subset of subjects.
**Follow up visits**	Monthly antenatal clinic visits and upon any other visits to the health centre for health concerns until delivery
**Outcome**	• adverse obstetric outcomes: abortion, perinatal mortality, stillbirth, preterm delivery, and unexplained neonatal death ≤7 days after birth<br>• adverse infant outcomes: congenital malformations regardless of the pregnancy outcome and neurological problems
**Ethical review**	The study was approved by the Rwandan National Ethics Committee prior to commencement. Each patient gave written informed consent before entry into the study.
**Notes**	• Some women were enrolled after delivery and were excluded from the IPD analysis (n=288+7)
**Bias assessment**	Overall low risk of bias (6/9 stars)<br>• Selection: 3 /4 stars - enrolled from 10 health facilities<br>• Comparability: 1 /2 stars - the distinction between the effects of malaria and AL exposure could not be made in this study (confounding by indication possible as unexposed not treated for malaria). Unexposed recruited from the same population contemporaneously.<br>• Outcome assessment: 3 /4 stars - the outcome was ascertained prospectively and retrospectively. Attrition was low: 20 women out of 2070 without complete data. Relatively late recruitment at ANC means only late miscarriages were captured.
**Citation**	Rulisa S, Kaligirwa N, Agaba S, Karema C, Mens PF, de Vries PJ. Pharmacovigilance of artemether-lumefantrine in pregnant women followed until delivery in Rwanda. c; 11: 225.[7]

Study Identifier	Mosha, Tanzania
**Investigator/Contact**	Dominque Mosha
**Country**	Tanzania
**Study Period**	April 2012-March 2013
**Study Design**	Multi-centre, prospective cohort study
**Participants**	Pregnant women were recruited from 22 Maternal Health clinics or from monthly house visits via demographic surveillance in the two HDSS sites
**Inclusion and Exclusion criteria**	Pregnant women with gestational age <20 weeks were recruited from the Reproductive and Child Health (RCH) clinic during their routine ANC visits and the community through monthly round-based house visits and routine HDSS quarterly census.
**Exposure ascertainment**	Self-report at ANC and verified by assessing patient's medical log in the attended health facility, prescription sheet and maternal RCH card.
**Gestational age measurements**	LMP or fundal height examination, when the LMP was unknown
**Follow up visits**	Women were followed every month until delivery to monitor pregnancy and birth outcomes.
**Outcome**	• Pregnancy Outcomes: maternal mortality, spontaneous abortion (pregnancy loss ≤ 28 weeks of gestation), stillbirth and live birth.<br>• Birth outcome included birth weight, maturity status at birth and presence of congenital anomalies (under the guidance of a specifically developed checklist).
**Ethical review**	Ethical approval was granted by the Ifakara Health Institute (IHI) ethical review board and the National Institute for Medical Research (NIMR) ethical committee. Written informed consent was obtained from all participants.
**Notes**	Issues with the recording of LMP- manually derived gestational age at the time of exposure but gestational age at the time of exit was unreliable
**Bias assessment**	Overall low risk of bias (6/9 stars)<br>• Selection: 3 /4 stars - enrolled from 22 ANC clinics and 20% from community<br>• Comparability: 1 /2 stars - confounding by indication possible as not randomised/blinded allocation of treatment. Unexposed recruited from the same population contemporaneously.<br>• Outcome assessment: 3 /4 stars – the outcome was ascertained prospectively. Attrition was low: 2167 pregnant women were recruited and 1783 (82·3%) completed the study until delivery. Relatively late recruitment at ANC means only late miscarriage were captured.
**Citation**	Mosha D, Mazuguni F, Mrema S, Sevene E, Abdulla S, Genton B. Safety of artemether-lumefantrine exposure in first trimester of pregnancy: an observational cohort. Malar J 2014; 13(1): 197.[8]

**Study Identifier**	Dellicour, Kenya
**Investigator/Contact**	Stephanie Dellicour
**Country**	Kenya
**Study Period**	February 2011- December 2013
**Study Design**	Prospective cohort study, part of multi-country ASAP protocol
**Participants**	Women of childbearing age (15-49 years) under enhanced morbidity surveillance
**Inclusion and Exclusion criteria**	Women between 15 and 49 years of age and active participants of an ongoing morbidity surveillance program under HDSS. Exclusion criteria included: inability to give informed consent or provide an accurate medical history.
**Exposure ascertainment**	Drug exposure data were captured using three approaches:<br>• interviews with pregnant women visiting the antenatal clinic in a referral health facility and at the time of pregnancy outcome follow-up;<br>• record linkage to data on drugs prescribed to WOCBA at the outpatient department in Lwak Hospital<br>• weekly to twice-monthly home visits by fieldworkers as part of an ongoing morbidity surveillance program
**Gestational age measurements**	LMP; ultrasound; fundal height and Ballard Score- assessment based on most accurate measure available
**Follow up visits**	Through the recommended ANC visit schedule and after pregnancy outcome
**Outcome**	Pregnancy outcomes captured at the health facility or at home included: miscarriages, stillbirths, live births, and major congenital malformations detectable at birth by surface examination.
**Ethical review**	The EMEP study was approved by the ethics committees and institutional review boards of CDC, KEMRI the Liverpool School of Tropical Medicine and the Institutional Review Board of the University of Washington. Written informed consent or assent was obtained from each participant.
**Notes**	Low agreement between data sources through record linkage, a high number of unconfirmed first trimester exposures and inability to assess the effect of the number of exposures on the outcome
**Bias assessment**	Overall low risk of bias (7/9 stars)<br>• Selection: 4 /4 stars - enrolled women from the community (low refusal rates)<br>• Comparability: 1 /2 stars - confounding by indication possible as not randomised/blinded allocation of treatment. Unexposed recruited from the same population contemporaneously.<br>• Outcome assessment: 3 /4 stars – the outcome was ascertained prospectively. Attrition was low: 3% (8/299) discontinued in the ACT exposure group and 4% (31/835) in the unexposed group before the end of pregnancy (3·4% overall).
**Citation**	1. Dellicour S, Desai M, Aol G, Oneko M, Ouma P, et al. Risks of miscarriage and inadvertent exposure to artemisinin derivatives in the first trimester of pregnancy: a prospective study in western Kenya. Malaria Journal 2015; 14: 461.[9]<br>2. Tinto H, Sevene E, Dellicour S, Macete E, d'Alessandro U, et al. Assessment of the Safety of Antimalarial Drug Use during Early Pregnancy: protocol for a multicenter prospective cohort study in Burkina Faso, Kenya and Mozambique. Reproductive Health 2015; 12: 112.[4]

**Study Identifier**	Sevene, Mozambique
**Investigator/Contact**	Esperanca Sevene
**Country**	Mozambique
**Study Period**	September 2011- June 2013
**Study Design**	Prospective cohort study, part of multi-country ASAP protocol
**Participants**	Pregnant women identified within the health demographic surveillance system (HDSS) or presenting at ANC (1 health facility)
**Inclusion and Exclusion criteria**	• Eligible participants consisted of pregnant women residing in the defined catchment areas, who planned to remain in the study area through delivery and who were willing and able to provide informed consent.<br>• Exclusion criteria were refusal to participate or be followed up at the end of pregnancy and any condition that would interfere with the ability to provide informed consent or provide an accurate medical history.
**Exposure ascertainment**	The ascertainment of drug exposure was multi-modal and included self-report (prospective and retrospective) and linkage to treatment records at local health facilities, drug prescribing and dispensing clinics.
**Gestational age measurements**	LMP; ultrasound; and Ballard Score- assessment based on most accurate measure available
**Follow up visits**	Through ANC visits and at delivery
**Outcome**	Pregnancy outcomes captured included: late miscarriages, stillbirths, live births, and major congenital malformations detectable at birth by surface examination.
**Ethical review**	The protocol was reviewed and approved by the National Bioethics Committee in Mozambique and the Institutional Review Board of the University of Washington.
**Notes**	
**Bias assessment**	Overall low risk of bias (6/9 stars)<br>• Selection: 3 /4 stars - enrolled from 4 ANC clinics<br>• Comparability: 1 /2 stars - confounding by indication possible as not randomised/blinded allocation of treatment. Unexposed recruited from the same population contemporaneously.<br>• Outcome assessment: 3 /4 stars – the outcome was ascertained prospectively. Attrition was low: 8·0% (2/25) discontinued in the ACT exposure group and 4·7% (35/738) in the unexposed group before the end of pregnancy. Relatively late recruitment at ANC means only late miscarriages were captured.
**Citation**	Tinto H, Sevene E, Dellicour S, Macete E, d'Alessandro U, et al. Assessment of the Safety of Antimalarial Drug Use during Early Pregnancy: protocol for a multicenter prospective cohort study in Burkina Faso, Kenya and Mozambique. Reproductive Health 2015; 12: 112.[4]

**Study Identifier**	Tinto, Burkina Faso
**Investigator/Contact**	Halidou Tinto
**Country**	Burkina Faso
**Study Period**	April 2011- December 2012
**Study Design**	Prospective cohort study, part of multi-country ASAP protocol
**Participants**	Pregnant women identified within the health and demographic surveillance system (HDSS) catchment area or presenting at ANC (1 health facility)
**Inclusion and Exclusion criteria**	• Eligible participants consisted of pregnant women residing in the defined catchment areas, who planned to remain in the study area through delivery and who were willing and able to provide informed consent.<br>• Exclusion criteria were refusal to participate or be followed up at the end of pregnancy and any condition that would interfere with the ability to provide informed consent or provide an accurate medical history.
**Exposure ascertainment**	The ascertainment of drug exposure was multi-modal and included self-report (prospective and retrospective) and linkage to treatment records at local health facilities, drug prescribing and dispensing clinics.
**Gestational age measurements**	LMP; ultrasound; and Ballard Score- assessment based on most accurate measure available
**Follow up visits**	Through ANC visits and at delivery
**Outcome**	Pregnancy outcomes captured included: late miscarriages, stillbirths, live births, and major congenital malformations detectable at birth by surface examination.
**Ethical review**	The protocol was reviewed and approved by the Ethical Review Boards of Centre Muraz Institutional Ethics committee and National Ethics committee in Burkina Faso, and the Institutional Review Board of the University of Washington.
**Notes**	
**Bias assessment**	Overall low risk of bias (6/9 stars)<br>• Selection: 3 /4 stars - enrolled from 4 ANC clinics<br>• Comparability: 1 /2 stars - confounding by indication possible as not randomised/blinded allocation of treatment in the first trimester. Unexposed recruited from participants of a randomised controlled trial.<br>• Outcome assessment: 3 /4 stars – the outcome was ascertained prospectively. Attrition was low: 0/ 42 discontinued in ACT exposure group and 2·4% (11/672) in the unexposed group before the end of pregnancy. Relatively late recruitment at ANC means only late miscarriages were captured.
**Citation**	Tinto H, Sevene E, Dellicour S, Macete E, d'Alessandro U, et al. Assessment of the Safety of Antimalarial Drug Use during Early Pregnancy: protocol for a multicenter prospective cohort study in Burkina Faso, Kenya and Mozambique. Reproductive Health 2015; 12: 112.[4]

**Study Identifier**	Rouamba, Burkina Faso
**Investigator/Contact**	Toussaint Rouamba
**Country**	Burkina Faso
**Study Period**	August 2012 to July 2014
**Study Design**	Prospective cohort study nested within an intervention study entitled 'ANC & Malaria Diagnostic in Pregnancy'
**Participants**	Pregnant women attending public health facilities.<br>Pregnant women were recruited at antenatal clinics and were monitored by study staff (midwives, nurses and physicians) at their scheduled ANC visits until delivery
**Inclusion and Exclusion criteria**	Eligible health centres are those that are:<br>• Located in the geographical location of Dafra district<br>• Have a minimum attendance of 200 pregnant women per year<br>• other public health facilities, private clinics and Dafra District Hospital will be excluded from the study
**Exposure ascertainment**	The ascertainment of drug exposure was multi-modal and included self-report (prospective and retrospective) and linkage to treatment records at local health facilities, drug prescribing and dispensing clinics.
**Gestational age measurements**	LMP; ultrasound; and Ballard Score- assessment based on most accurate measure available
**Follow up visits**	Through ANC visits and at delivery
**Outcome**	Pregnancy outcomes captured included late miscarriages, stillbirths, live births, and major congenital malformations detectable at birth by surface examination.
**Ethical review**	The study was approved by the ethics committee of Centre Muraz, Bobo Dioulasso, Burkina Faso and the National ethics committee of Burkina Faso, and the WHO Ethics Review Committee,
**Notes**	Information on neonates with serious birth defects will be reviewed by an international birth defects panel, coordinated by WHO for verification of diagnosis and advice (where requested) on the management of the case
**Bias assessment**	Overall low risk of bias (6/9 stars)<br>• Selection: 3 /4 stars - enrolled from 8 ANC clinics<br>• Comparability: 1 /2 stars - confounding by indication possible as not randomised/blinded allocation of treatment. Unexposed recruited from the same population contemporaneously.<br>• Outcome assessment: 3 /4 stars - outcome was ascertained prospectively.
**Citation**	Rouamba T, Valea I, Bognini JD, Kpoda H, Mens PF, Gomes MF, Tinto H, Kirakoya-Samadoulougou F. Safety Profile of Drug Use During Pregnancy at Peripheral Health Centres in Burkina Faso: A Prospective Observational Cohort Study. Drugs Real World Outcomes. 2018; 5(3):193-206.[3]

**Study Identifier**	WHO / the Special Programme for Research and Training in Tropical Diseases (TDR), multi-country
**Investigator/Contact**	Melba Gomes
**Country**	Tanzania, Uganda, Kenya and Ghana
**Study Period**	2010-2011
**Study Design**	Prospective cohort study at sentinel Health Centres where women came for antenatal care and were likely to come for delivery.
**Participants**	Pregnant women attending public health facilities.<br>Pregnant women were recruited at antenatal clinics and were monitored by study staff (midwives, nurses and physicians) at their scheduled ANC visits until delivery<br>A local paediatrician/physician's commitment was required to review births and photographs of births and advise on the management of infants found with serious birth complications
**Inclusion and Exclusion criteria**	• Inclusion – all pregnant women presenting for the first time during the pregnancy at antenatal care<br>• Moderate to high prevalence of malaria in the setting<br>• Informed consent to be included in the Pregnancy Registry
**Exposure ascertainment**	One or more medical records confirming treatment for malaria during the pregnancy<br>Self-report (prospective and retrospective) of medication was linked with treatment records at local health facilities, drug prescribing and dispensing clinics.
**Gestational age measurements**	LMP
**Follow up visits**	Through ANC visits and at delivery. If the woman did not deliver or come to any ANC visit as scheduled, she was followed up through phone calls or visits to the home.
**Outcome**	Pregnancy outcomes captured included: late miscarriages, stillbirths, live births, and major congenital malformations detectable at birth by surface examination.
**Ethical review**	WHO Ethics Review Committee
**Notes**	This study is regarded as four cohorts in the analysis.
**Bias assessment**	Overall low risk of bias (7/9 stars)<br>• Selection: 3 /4 stars - enrolled from several health facilities (2 in each country)<br>• Comparability: 1 /2 stars – malaria was not always diagnosed through parasitology (microscopy or RDTs). Therefore, an unequivocal difference between presumed malaria and confirmed exposure could not be made.<br>• Outcome assessment: 3 /4 stars - the outcome was ascertained prospectively. Attrition was low. Miscarriage, even late miscarriage, could not be captured reliably.
**Citation**	Mehta U, Clerk, C., Allen, C., Yore M., Sevene E., Singlovic J., Mangiaterra V., Elefant E., Sullivan F., Holmes L., Gomes M. Protocol for a drugs exposure pregnancy registry for implementation in resource-limited settings. BMC Pregnancy and Childbirth 2012, 12: 89.[2]

**Study Identifer**	McGready, Thailand-Myanmar
**Author/ Principal Investigator**	Rose McGready
**Country**	Thailand-Myanmar border
**Study Period**	2000-2017
**Study Design**	Prospective cohort study at Health facilities where women came for antenatal care. Some participants were also enrolled in a controlled trial.
**Participants**	Pregnant women attending health facilities on the Thailand-Myanmar border.
**Inclusion and Exclusion criteria**	• Inclusion – all pregnant women presenting to antenatal clinics during their first trimester with a viable fetus.
**Exposure ascertainment**	Women were screened for malaria, and data on malaria, antimalarial treatment, and birth outcomes were collected. One or more medical records confirming treatment for malaria during the pregnancy
**Gestational age measurements**	Ultrasound (or LMP, Dubowitz)
**Follow up visits**	Through ANC visits and at delivery.
**Outcome**	Pregnancy outcomes captured included: miscarriages, stillbirths, live births, and major congenital anomalies detectable at birth by surface examination and chest auscultation.
**Ethical review**	The Oxford Tropical Research Ethics Committee granted ethical, and the Tak Province Community Ethics Advisory Board provided local permission
**Notes**	The previous pooled analysis included data before 2000 when gestational age was not estimated by ultrasound.
**Bias assessment**	Overall low risk of bias (7/9 stars)<br>• Selection: 3 /4 stars -all pregnant women enrolled from several health facilities<br>• Comparability: 1 /2 stars – confounding by indication possible as not randomised/blinded allocation of treatment in the first trimester. Unexposed recruited from the same population contemporaneously. Malaria was diagnosed through parasitology (microscopy or RDTs).<br>• Outcome assessment: 3 /4 stars – the outcome was ascertained prospectively. Attrition was low. Early miscarriage could not be captured reliably.
**Citation**	1. Moore KA, Simpson JA, Wiladphaingern J, et al. Influence of the number and timing of malaria episodes during pregnancy on prematurity and small-for-gestational-age in an area of low transmission. BMC Med 2017; 15(1): 117.[10]<br>2. Moore KA, Simpson JA, Paw MK, Pimanpanarak M, Wiladphaingern J, Rijken MJ, et al. Safety of artemisinins in first trimester of prospectively followed pregnancies: an observational study. Lancet Infect Dis. 2016;16:576–83.[11]<br>3. Saito M, Carrara VI, Gilder ME, et al. A randomized controlled trial of dihydroartemisinin-piperaquine, artesunate-mefloquine and extended artemether-lumefantrine treatments for malaria in pregnancy on the Thailand-Myanmar border. BMC Med. 2021; 19(1): 132.[12]

Table 2. Descriptions of articles reporting first trimester exposures but excluded from the meta-analysis.

Study	Country	Study Period	Population Source	Study design	Antimalarials exposure 1st trimester	Comparison group	Findings 1st Trimester	Reason for exclusion
**Studies excluded because they don't meet the eligibility criteria (n=7)**								
Adam, 2004[13]	Sudan	1997-2001	Pregnant women who presented with symptoms of P. falciparum malaria and had confirmed malaria parasites were treated with quinine and returned to the hospital with recurrent malaria symptoms and parasite detected within three weeks.	Case series	Art Par.= 1	N/A	No abortion, stillbirth or congenital abnormalities in the newborn baby exposed in-utero to artemether.	Not meeting eligibility criteria: no internal comparator
Adam, 2009[14]	Sudan	2006–2008	Pregnant women in the first or second trimester who were attending antenatal-care clinics were asked if they had had malaria in the first trimester of the index pregnancy and the women who had received artemisinins were followed-up until delivery, and their babies were followed-up until they were 1-year-olds.	Case series	ASSP=11, Art Par.=48, AL=3	N/A	Two miscarriages among women receiving artemether injection who also received quinine for a second attack. The other women delivered healthy babies at full term. No congenital malformations, no preterm labour, no maternal deaths; none of the babies died during their first year of life.	Not meeting eligibility criteria: no internal comparator
Ahmed, Unpublished[15]	Indonesia	2018-19	Women enrolled during antenatal care at the study health facilities and followed up to cover delivery and post-natal period of 8 weeks. Self-reported malaria treatment in early pregnancy or close to the time before becoming aware of current pregnancy was recorded.	Prospective cohort	DP=45 (before 24 weeks)	Unexposed=41	All pregnancies treated before 24 weeks had a livebirth. There were no congenital anomalies	Not meeting eligibility criteria: no internal comparator
	Indonesia	2006-17	Pregnant women identified through health facility registers (delivery units, pharmacy, emergency, outpatient/inpatient and laboratory records) at 2 selected hospitals	Retrospective record linkage study cohort	DP=159	Qui=636	No difference between DHP treatment and quinine treatment in the first trimester (Hazard Ratio 1·00, 95% CI 0·32, 3·14; p=0·997)<br>Quinine: 12 (1·9%) abortions, 3 (0·5%) stillbirths and no congenital anomalies<br>DHP: 3 (1·9%) abortions, 1 (0·6% stillbirth) and no congenital anomalies	Not meeting eligibility criteria: retrospective study design

Study	Country	Study Period	Population Source	Study design	Antimalarials exposure 1st trimester	Comparison group	Findings 1st Trimester	Reason for exclusion
Deen, 2001[16]	The Gambia	1999-2000	All women of reproductive age (15–44 years) residing in the 42 study villages. Villages were part of a mass drug administration campaign. Pregnant women exposed to Mass drug administration were identified retrospectively.	Retrospective cohort	ASSP =77	Placebo = 40 Unexposed = 132	No evidence of a teratogenic effect, no evidence of increased foetal loss or infant death	Not meeting eligibility criteria: retrospective design women recruited after pregnancy outcome
Dellicour, 2013[17]	Senegal	2004–08	Record linkage study of women attending ANC and deliveries in Mlomp Dispensary	Retrospective cohort record linkage	ASAQ=7	NA	Exposure to ACTs resulted in normal live births with no congenital anomalies.	Not meeting eligibility criteria: no internal comparator
Poespoprodjo, 2014 [18]	Indonesia	2004- 2009	All pregnant women and newborn infants admitted to the maternity ward were screened for malaria.	Prospective cohort	DP =8 DP+ivAS =5 AS=5	iv Qui = 50, oral Qui=38	The risk of abortion was over 60% (5/8) in women receiving an ACT compared to 1% (1/38) in women treated with quinine. None of the 10 women who received IV artesunate miscarried.	Not included in the meta-analysis as high risk of bias by the severity of symptoms where women with more severe symptoms were treated with the drug suspected to have better efficacy (DP rather than Q).
Rouamba, 2020[19]	Burkina Faso	2010–12	Active pharmacovigilance surveillance in HDSS. Patients treated with ACTs were followed prospectively	Prospective cohort	ASAQ=13	NA	12 women delivered live newborns (including one with twins) with no congenital malformations. One woman had experienced a spontaneous abortion with a birth defect (a type of cervical agenesis and defect of the dome of the skull) that was judged not to be related to ASAQ as it occurred 4 days after exposure.	Not meeting eligibility criteria: no internal comparator
**Publications excluded because of overlap with the pooled Thailand-Myanmar cohort included in the analysis**								
McGready, 2001[20]	Thailand-Myanmar border	1986–2001	Pregnant women with microscopy confirmed *P. falciparum* or mixed *P. falciparum* and *P. vivax* infections.	Prospective cohort	Artesunate or artemether alone or in combination with MQ, C AP, or artesunate iv, or AL 1st trim = 40	Unexposed = 8154	The rates of abortion, congenital abnormality, and stillbirth were all within the normal range of their communities	Overlap in study period between the different SMRU publications (McGready and Moore)[11,21]

Study	Country	Study Period	Population Source	Study design	Antimalarials exposure 1st trimester	Comparison group	Findings 1st Trimester	Reason for exclusion
McGready, 2012[21]	Thailand-Myanmar border	1986-2010	Pregnant women in camps for refugees with uncomplicated, multidrug-resistant *P. falciparum* malaria.	Prospective cohort	AS=64, Qui=355, CQ=354		There were no significant differences in the rates of miscarriage for artesunate, quinine, and chloroquine following treatment during the first trimester (rates of miscarriage were 31% (20/64), 27% (95/355), and 26% (92/354), respectively)	
Moore et al., 2016[11]	Thailand-Myanmar border	1994-2013	Pregnant women living in refugee camps with *P. falciparum* malaria transmission.	Prospective cohort	AS=99, MQAS=71, AL=10, DP=3		No evidence that first-line treatment with an artemisinin derivative was associated with an increased risk of miscarriage or congenital malformations	

AL, artemether-lumefantrine, ASAQ, amodiaquine-artesunate; Art Par., parenteral artemether, ASSP; artesunate-sulfadoxine-pyrimethamine; AP, atovaquone-proguanil; DP; dihydroartemisinin-piperaquine; C, clindamycin; iv, intravenous; MQ-AS, mefloquine-artesunate, MQ, mefloquine
Summary of exposures from excluded studies (not counting SMRU data included in the updated meta-analysis): AL=3, DP=217, ASSP=88, ASAQ=20, parenteral artemisinins=59

Table 3. Characteristics of the studies included in the IPD meta-analysis.

Study	County	Study period	No. confirmed ABT	No. confirmed non ABT	No. unexposed	Mean gestational age at enrollment (SD)	Mean weeks of follow-up (SD)	Mean maternal age (SD)	Primigravida (%)
Rouamba[3]	Burkina Faso	2012-2015	13	152	4354	16·9 (6·6)	19·3 (6·9)	25·3 (5·9)	1095/4514 (24%)
WHO TDR[2]	Ghana	2010-2011	5	4	246	15·6 (6·8)	21·7 (8·0)	27·1 (5·0)	70/254 (28%)
	Kenya	2010-2011	3	3	230	25·2 (3·9)	13·9 (4·4)	24·7 (5·0)	77/235 (33%)
	Tanzania	2010-2011	0	0	187	18·1 (6·5)	17·3 (7·2)	27·8 (5·6)	37/183 (20%)
	Uganda	2011-2012	3	3	171	26·9 (6·7)	11·4 (6·3)	24·7 (4·1)	63/177 (36%)
Mosha[8]	Tanzania	2012-2013	156	69	1527	14·7 (3·5)	22·0 (4·5)	25·8 (6·8)	872/1744 (50%)
Dellicour[4,9]ǂ	Kenya	2011-2013	71	2	1075	15·9 (9·9)	19·8 (11·0)	25·7 (6·8)	219/1099 (20%)
Sevene[4]ǂ	Mozambique	2011-2013	19	5	710	21·0 (5·7)	16·6 (6·5)	24·2 (6·2)	383/732 (52%)
Tinto[4]ǂ	Burkina Faso	2011-2013	30	21	626	24·0 (6·2)	14·6 (6·3)	27·0 (6·6)	228/677 (34%)
Rulisa[7]	Rwanda	2007-2009	77	0	1571	28·0 (7·5)	11·2 (7·1)	27·5 (5·7)	747/1576 (47%)
Manyando[6]	Zambia	2004-2008*	166	6	763	24·8 (8·0)	13·6 (7·9)	25·2 (5·6)	450/935 (48%)
McGready[11,12,21]	Thailand-Myanmar	2000-2017	194	811	20905	9·1 (2·6)	25·1 (9·8)	26·3 (6·7)	5862/21910(27%)

ǂ counted as one study.[4]

*categorised as 2005-2009 in the analyses because individual patient-level data on the exact date (year) were not available, and this study started in October 2004.

ABT: artemisinin-based treatment. SD: standard deviation

3. Baseline characteristics for analyses restricted to embryo-sensitive period analyses, and analyses comparing artemether-lumefantrine and oral quinine

Table 4. Characteristics of women with confirmed exposure to artemisinin or non-artemisinin in the embryo-sensitive period and those unexposed in the embryo-sensitive period

	Unexposed (n=32947)		Artemisinin-exposed* (n=584)		Non-artemisinin-exposed** (n=823)	
	N	Mean (SD)/% (n)	N	Mean (SD)/% (n)	N	Mean (SD)/% (n)
EGA at exposure		NA	584	9·1 (2·1)	823	9·5 (2)
Duration of follow-up	32947	22·4 (9·9)	584	20·7 (9)	823	24·0 (10)
Pregnancy outcome available	32947	88·7 (29210)	584	93·2 (544)	823	74·1 (610)
**Baseline characteristics**						
Age (years)	32849	26.1 (6.5)	584	25.8 (6.4)	823	24.9 (6.4)
Gravidity	32805		584		822	
1		29·3 (9619)		44·7 (261)		31·4 (258)
2		20·8 (6810)		16·1 (94)		19·6 (161)
≥3		49·9 (16376)		39·2 (229)		49·0 (403)
Parity	27811		180		783	
0		29·9 (8303)		38·9 (70)		36·3 (284)
1		22·8 (6353)		21·1 (38)		19·8 (155)
≥2		47·3 (13155)		40·0 (72)		43·9 (344)
Previous miscarriage (Yes)	28838	21·5 (6199)	412	14·8 (61)	794	26·6 (211)
Previous stillbirth (Yes)	28257	2·7 (772)	410	2·0 (8)	749	4·1 (31)
Height (m)	1494	1·6 (0·1)	63	1·6 (0·1)	5	1·6 (0·1)
Body weight (kg)	1523	59·8 (9·5)	160	50·4 (10)	617	46·2 (6·2)
Marital status: married	25178	96·8 (24362)	356	88·2 (314)	689	99·6 (686)
HIV (positive)	8038	8·4 (673)	433	6·2 (27)	131	3·1 (4)
Smoking: yes	23242	21·0 (4892)	206	18·0 (37)	646	36·4 (235)
Alcohol: yes	2156	14·1 (304)	84	10·7 (9)	18	33·3 (6)
Literate	8519	62·5 (5321)	40	50·0 (20)	86	46·5 (40)
Education	4827		370		57	
No		19·8 (956)		14·6 (54)		7·0 (4)
primary		61·2 (2954)		58·6 (217)		75·4 (43)
secondary or higher		19·0 (917)		26·8 (99)		17·5 (10)
IPTp doses	7130		99		140	
0		22·0 (1569)		37·4 (37)		19·3 (27)
1		31·7 (2257)		21·2 (21)		34·3 (48)
2		38·7 (2761)		24·2 (24)		42·1 (59)
3		5·9 (418)		13·1 (13)		4·3 (6)
4		1·8 (125)		4·0 (4)		0 (0)
N/A	21158		122		638	
Gestational age by ultrasound	29357	69·2 (20318)	355	46·5 (165)	783	63·5 (497)
Country	32947		584		823	
Burkina Faso		15·5 (5093)		5·1 (30)		16·0 (132)
Ghana		0·8 (250)		0·5 (3)		0·4 (3)
Kenya		4·3 (1419)		8·4 (49)		0·5 (4)

	Unexposed (n=32947)		Artemisinin-exposed* (n=584)		Non-artemisinin-exposed** (n=823)	
	N	Mean (SD)/% (n)	N	Mean (SD)/% (n)	N	Mean (SD)/% (n)
Tanzania		5·3 (1761)		26·4 (154)		4·0 (33)
Uganda		0·5 (181)		0·5 (3)		0·4 (3)
Mozambique		2·2 (711)		3·4 (20)		0·5 (4)
Rwanda		4·8 (1577)		12·2 (71)		0 (0)
Zambia		2·4 (797)		22·6 (132)		0·7 (6)
Thailand-Myanmar		64·2 (21158)		20·9 (122)		77·5 (638)
Study year	32947		584		823	
2000–2004		16·2 (5349)		6·5 (38)		35·0 (288)
2005–2009		30·7 (10131)		42·3 (247)		35·8 (295)
2010–2017		53·0 (17467)		51·2 (299)		29·2 (240)

EGA: estimated gestational age; IPTp: intermittent preventive treatment in pregnancy; N: number of women evaluated; N/A: not applicable; SD: standard deviation.

Women are categorised according to the first exposure in the embryo-sensitive period. Unexposed women here represent pregnancies that had no antimalarial exposure according to any sources during the embryo-sensitive period.

* Including 512 ACT (445 AL, 22 ASAQ, 29 ASMQ, 14 DP, 2 artesunate-atovaquone-proguanil), 69 AS/AC, 3 parenteral artesunate based on first exposure in the embryo-sensitive period.

** including 686 oral quinine (514 quinine monotherapy, 172 quinine+clindamycin), 9 parenteral quinine, 128 chloroquine based on first exposure in the embryo-sensitive period.

Table 5. Characteristics of women with confirmed exposure to artemether-lumefantrine or oral quinine in the first trimester and those unexposed in the first trimester

	Unexposed (n=32470)		Artemether-Lumefantrine (n=525)		Oral Quinine (n=917)	
	**N**	**Mean (SD)/% (n)**	**N**	**Mean (SD)/% (n)**	**N**	**Mean (SD)/% (n)**
EGA at exposure		NA	525	8·6 (2·7)	917	9·0 (3)
Duration of follow-up	32470	22·4 (9·9)	525	20·0 (8·5)	917	22·5 (10·6)
Pregnancy outcome available	32470	88·4 (28698)	525	97·5 (512)	917	67·9 (623)
**Baseline characteristics**						
Age (years)	32373	26.1 (6.5)	525	25.8 (6.1)	917	25.3 (6.6)
Gravidity	32331		524		915	
1		29·4 (9494)		46·9 (246)		29·6 (271)
2		20·8 (6709)		15·3 (80)		19·9 (182)
≥3		49·9 (16128)		37·8 (198)		50·5 (462)
Parity	27519		55		840	
0		29·9 (8220)		43·6 (24)		33·6 (282)
1		22·8 (6278)		23·6 (13)		19·6 (165)
≥2		47·3 (13021)		32·7 (18)		46·8 (393)
Previous miscarriage (Yes)	28402	21·5 (6100)	329	7·3 (24)	873	27·6 (241)
Previous stillbirth (Yes)	27824	2·7 (759)	329	0·3 (1)	829	4·3 (36)
Height (m)	1415	1·6 (0·1)	83	1·6 (0·1)	7	1·6 (0·1)
Body weight (kg)	1538	58·9 (9·9)	104	55·6 (10·1)	629	46·4 (6·8)
Marital status: married	24868	96·8 (24079)	260	81·5 (212)	757	98·8 (748)
HIV (positive)	7778	8·3 (643)	478	7·3 (35)	188	2·7 (5)
Smoking: yes	22995	21·1 (4842)	107	2·8 (3)	677	37·1 (251)
Alcohol: yes	2058	14·3 (295)	84	1·2 (1)	28	32·1 (9)
Literate	8500	62·5 (5316)	16	56·3 (9)	74	44·6 (33)
Education	4635		408		102	
No		19·6 (907)		15·9 (65)		5·9 (6)
primary		61·4 (2846)		55·4 (226)		72·5 (74)
secondary or higher		19·0 (882)		28·7 (117)		21·6 (22)
IPTp doses	6905		97		177	
0		21·8 (1504)		39·2 (38)		21·5 (38)
1		31·9 (2204)		16·5 (16)		33·3 (59)
2		39·0 (2693)		19·6 (19)		39·5 (70)
3		5·6 (390)		18·6 (18)		5·1 (9)
4		1·7 (114)		6·2 (6)		0·6 (1)
N/A	21006		22		661	
Gestational age by ultrasound	28934	69·7 (20155)	288	27·4 (79)	842	59·6 (502)
Country	32470		525		917	
Burkina Faso		15·3 (4983)		1·3 (7)		18·5 (170)
Ghana		0·8 (247)		0·6 (3)		0·4 (4)
Kenya		4·0 (1305)		14·1 (74)		0·2 (2)
Tanzania		5·3 (1714)		29·7 (156)		7·5 (69)
Uganda		0·5 (171)		0·2 (1)		0 (0)
Mozambique		2·2 (710)		3·6 (19)		0·5 (5)
Rwanda		4·8 (1571)		14·7 (77)		0 (0)

	Unexposed (n=32470)		Artemether-Lumefantrine (n=525)		Oral Quinine (n=917)	
	**N**	**Mean (SD)/% (n)**	**N**	**Mean (SD)/% (n)**	**N**	**Mean (SD)/% (n)**
Zambia		2·3 (763)		31·6 (166)		0·7 (6)
Thailand-Myanmar		64·7 (21006)		4·2 (22)		72·1 (661)
Study year	32470		525		917	
2000–2004		16·2 (5274)		0·8 (4)		34·5 (316)
2005–2009		30·9 (10027)		46·5 (244)		33·2 (304)
2010–2017		52·9 (17169)		52·8 (277)		32·4 (297)

EGA: estimated gestational age; IPTp: intermittent preventive treatment in pregnancy; N: number of women evaluated; N/A: not applicable; SD: standard deviation.

Women are categorised according to the first exposure in the first trimester. Unexposed women here represent pregnancies that had no antimalarial exposure according to any sources during the first trimester.

4. Summary of primary analyses results using the unexposed women as the reference group

A

Outcome	Exposed	Unexposed	HR (95%CI)	aHR (95%CI)	p-value	E-value
Composite outcome						
First trimester	42/737	2393/32365	0.93 (0.68-1.27)	0.92 (0.67-1.26)	0.617	2.36
Embryo-sensitive period	37/584	2454/32947	1.07 (0.77-1.50)	1.03 (0.74-1.44)	0.849	2.07
Miscarriage						
First trimester	27/670	1807/30300	0.97 (0.65-1.44)	0.96 (0.65-1.43)	0.853	2.49
Embryo-sensitive period	23/533	1859/30849	1.15 (0.75-1.77)	1.09 (0.71-1.68)	0.692	2.20
Stillbirth						
First trimester	13/646	404/27947	0.88 (0.50-1.54)	0.84 (0.48-1.48)	0.556	3.63
Embryo-sensitive period	12/518	412/28428	0.95 (0.53-1.71)	0.91 (0.51-1.64)	0.762	3.39
Fetal loss						
First trimester	40/737	2211/32365	0.89 (0.64-1.23)	0.88 (0.64-1.22)	0.447	2.54
Embryo-sensitive period	35/584	2271/32947	1.01 (0.72-1.43)	0.97 (0.69-1.37)	0.863	2.30
Major congenital anomaly						
First trimester	2/737	182/32365	0.97 (0.24-3.97)	0.99 (0.24-4.03)	0.984	7.84
Embryo-sensitive period	2/584	183/32947	1.44 (0.35-5.91)	1.39 (0.34-5.71)	0.647	5.41

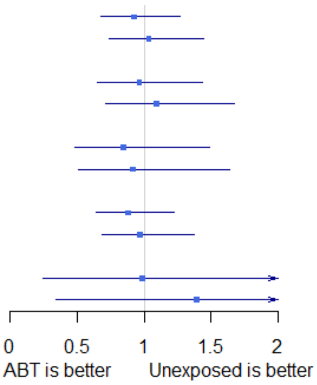

B

Outcome	Exposed	Unexposed	HR (95%CI)	aHR (95%CI)	p-value	E-value
Composite outcome						
First trimester	96/1076	2393/32365	1.32 (1.07-1.61)	1.30 (1.06-1.60)	0.013	NA
Embryo-sensitive period	60/823	2454/32947	1.07 (0.83-1.38)	1.08 (0.84-1.40)	0.548	1.71
Miscarriage						
First trimester	76/1072	1807/30300	1.32 (1.05-1.67)	1.30 (1.03-1.64)	0.027	NA
Embryo-sensitive period	46/819	1859/30849	1.05 (0.79-1.41)	1.07 (0.80-1.44)	0.638	1.84
Stillbirth						
First trimester	12/745	404/27947	1.19 (0.67-2.11)	1.19 (0.67-2.12)	0.549	2.38
Embryo-sensitive period	6/609	412/28428	0.78 (0.35-1.76)	0.78 (0.35-1.74)	0.537	5.30
Fetal loss						
First trimester	88/1076	2211/32365	1.29 (1.04-1.59)	1.27 (1.02-1.57)	0.031	NA
Embryo-sensitive period	52/823	2271/32947	1.00 (0.76-1.32)	1.01 (0.77-1.33)	0.951	1.97
Major congenital anomaly						
First trimester	8/1076	182/32365	1.63 (0.80-3.31)	1.65 (0.81-3.36)	0.171	1.82
Embryo-sensitive period	8/823	183/32947	1.87 (0.92-3.80)	1.92 (0.94-3.92)	0.074	1.36

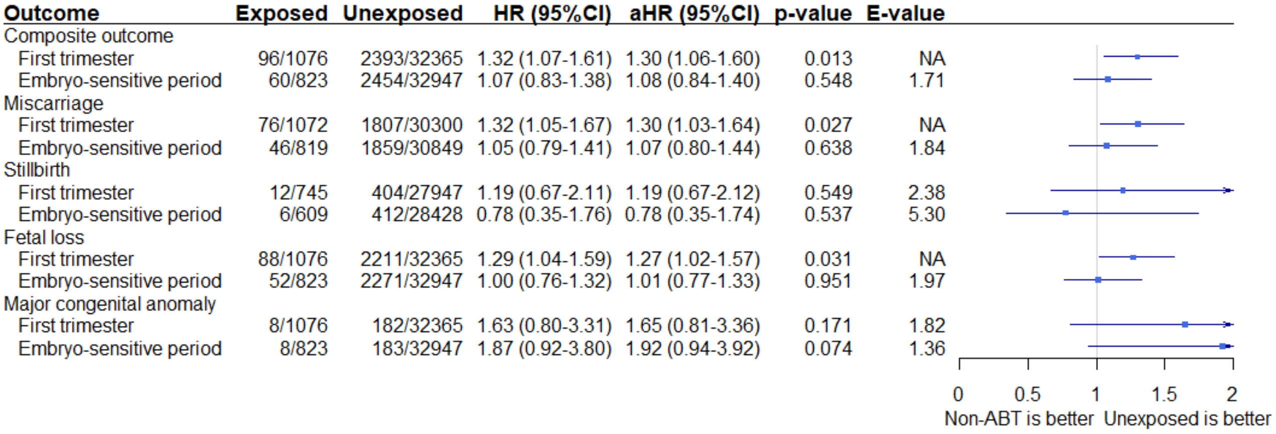

Figure 1. Adjusted hazard ratio of adverse pregnancy outcomes in the first trimester and during the embryo-sensitive period. (A) compares women treated with artemisinin-based treatment (ABT) and unexposed women (reference). (B) compares women treated with an antimalarial not containing an artemisinin (Non-ABT) and unexposed women (reference).

The composite primary outcome includes miscarriage or stillbirth, or major congenital anomalies.

Fetal loss includes miscarriage or stillbirth

Acronyms: aHR: adjusted hazard ratio, CI: confidence interval

Adjusted by age group (<20, 20s, 30s, >=40), gravidity (1, 2, >=3), and study year (2000–4, 2005–9, 2010–17). Shared frailty Cox model was fitted to adjust for within study clustering

Note: An E-value is the minimum risk ratio that an unmeasured confounder would need to have with the outcome and ABT-exposure to unmask an increased risk of adverse outcome in the women who were exposed to ABT (i.e., for the lower confidence interval to shift over the null, HR>1)

The numbers represent the pregnancies included in the unadjusted analysis. In the adjusted analysis, women (1 artemether-lumefantrine, 2 quinine and 162 unexposed) with a missing covariate were not included.

5. Description of major congenital anomalies by EUROCAT sub-groups

Table 6. Major congenital anomaly cases by EUROCAT0F[22] subgroups and exposure status for singleton live-births.

EUROCAT Subgroup	First trimester			Embryo-sensitive period	
	Unexposed (n=26270)	ABT (n=623)	Non ABT (n=681)	ABT (n=503)	Non ABT (n=558)
**All anomalies ***	182	2	8	2	8
**Nervous system**	27	0	0	0	0
Neural Tube Defects	17	0	0	0	0
Anencephalus and similar	8	0	0	0	0
Encephalocele	3	0	0	0	0
Spina Bifida	6	0	0	0	0
Hydrocephalus	5	0	0	0	0
Severe microcephaly	3	0	0	0	0
Arhinencephaly /holoprosencephaly	1	0	0	0	0
**Eye**	8	0	0	0	0
Anophthalmos /microphthalmos	8	0	0	0	0
Anophthalmos	7	0	0	0	0
Congenital cataract	1	0	0	0	0
**Ear, face and neck**	13	0	0	0	0
Anotia	6	0	0	0	0
**Congenital heart defects**[1]	15	0	1	0	1
Severe CHD	2	0	0	0	0
Tetralogy of Fallot	1	0	0	0	0
Hypoplastic left heart	1	0	0	0	0
**Oro-facial clefts**	30	1	2	0	2
Cleft lip with or without cleft palate	21	1	2	0	2
Cleft palate	9	0	0	0	0
**Digestive system**	20	0	0	1	0
Duodenal atresia or stenosis	2	0	0	0	0
Ano-rectal atresia and stenosis	12	0	0	1	0
Hirschsprung's disease	1	0	0	0	0
Diaphragmatic hernia	3	0	0	0	0
Abdominal wall defects	10	0	0	0	0
Gastroschisis	5	0	0	0	0
Omphalocele	4	0	0	0	0
**Urinary**	4	0	0	0	0
Congenital hydronephrosis	1	0	0	0	0

Table 6. Major congenital anomaly cases by EUROCAT0F[22] subgroups and exposure status for singleton live-births.

EUROCAT Subgroup	First trimester			Embryo-sensitive period	
	Unexposed (n=26270)	ABT (n=623)	Non ABT (n=681)	ABT (n=503)	Non ABT (n=558)
Posterior urethral valve and / or prune belly	1	0	0	0	0
**Genital**	8	0	0	0	0
Indeterminate sex	5	0	0	0	0
**Limb**	46	1	5	1	5
Limb reduction defects	7	0	0	0	0
Club foot – talipes equinovarus	27	0	3	0	3
Hip dislocation and /or dyspasia	1	0	0	0	0
Syndactyly	15	1	3	1	3
**Other anomalies /syndromes**	24	0	1	0	1
Skeletal dysplasias	1	0	0	0	0
Craniosynostosis	2	0	0	0	0
Congenital constriction bands / amniotic band	6	0	1	0	1
Congenital skin disorders	3	0	0	0	0
Vascular disruption anomalies	17	0	1	0	0
Teratogenic syndromes with malformations	1	0	0	0	0
Maternal infections resulting in malformations	1	0	0	0	0
Cases with anomalies excluded from EUROCAT subgroups[2]	3	0	0	0	0

* The number in different subgroups of anomalies cannot be added to reach a total number of cases with congenital anomalies as a baby with several anomalies is counted once within each subgroup of anomaly.

[1] 13 out of 16 cases were detected on the Thailand-Myanmar border, the only site systematically screening for heart murmurs. The cases included 1 fatal case, 4 cases with a heart murmur and other major anomalies, 5 cases with cyanosis, 6 cases with confirmed diagnosis: dysplastic pulmonary valve, teratology of Fallot, congenital atrioventricular block with a heart murmur, pulmonary artery atresia, hypoplastic left heart syndrome, ectopia cardis.

[2] Two cases with inguinal hernia and one case with fetal hydrops.

6. Subgroup analyses

6-1. Subgroup analyses by antimalarial type

Table 7. Number of exposure and outcomes for each confirmed artemisinin-based treatment in the first trimester or embryo-sensitive period

Outcome	ABT*	ACT	AL	ASAQ	ASMQ	DP	AS/AC
**First trimester (confirmed exposure)**							
Composite outcome	42/737	31/638	25/525	0/32	5/58	1/20	10/97
Miscarriage	27/670	20/571	15/465	0/25	4/58	1/20	7/97
Stillbirth	13/646	11/581	10/488	0/31	1/42	0/17	1/63
Fetal loss	40/737	31/638	25/525	0/32	5/58	1/20	8/97
Major congenital abnormality	2/737	0/638	0/525	0/32	0/58	0/20	2/97
**Embryo-sensitive period (confirmed exposure)**							
Composite outcome	37/584	29/512	22/445	0/22	6/29	1/14	8/71
Miscarriage	23/533	17/461	12/398	0/18	4/29	1/14	6/71
Stillbirth	12/518	11/473	10/415	0/21	1/22	0/13	1/44
Fetal loss	35/584	28/512	22/445	0/22	5/29	1/14	7/71
Major congenital abnormality	2/584	1/512	0/445	0/22	1/29	0/14	1/71

*ABT includes ACT, AS/AC and parenteral artemisinin-based treatments.
Women who were exposed to different ABT/ACTs are counted once for each group. Thus, pregnancies can contribute multiple times to the different columns, and the totals may differ from those reported in the other analyses that categorised pregnancies based on the first ABT exposure only and censored women from the next exposure onwards if this involved another treatment group (e.g. ABT vs non-ABT or ACT vs non-ACT).
One woman exposed to AS and then to DP (an ACT) was included in the ABT group, but excluded from the ACT-exposed group as she was censored at the first ABT (non-ACT) exposure.
ABT: artemisinin-based treatment. ACT: artemisinin-based combination therapies. AL: artemether-lumefantrine. ASAQ: artesunate-amodiaquine. ASMQ: artesunate-mefloquine. DP: dihydroartemisinin-piperaquine. AS/AC: artesunate monotherapy/artesunate-clindamycin.

Table 8. Adjusted hazard ratio of adverse pregnancy outcomes in women with confirmed exposure to oral artemisinin-based combination therapies (ACTs) or artemether-lumefantrine (AL) compared with women with confirmed exposure to oral non-artemisinin antimalarials in the first trimester for different types of exposures

	ACT* vs oral non-artemisinins					AL vs oral non-artemisinins				
	ACT	non-ABT	aHR (95%CI)	p-value	E-value	AL	non-ABT	aHR (95%CI)	p-value	E-value
Composite	31/636	96/1064	0·59 (0·39-0·89)	0·013	4·63	25/524	96/1064	0·64 (0·41-1·02)	0·058	4·41
Miscarriage	20/569	76/1062	0·64 (0·39-1·07)	0·092	4·67	15/464	76/1062	0·77 (0·42-1·39)	0·387	4·19
Stillbirth	11/579	12/733	0·61 (0·27-1·40)	0·244	7·08	10/488	12/733	0·60 (0·26-1·42)	0·248	7·33
Fetal loss	31/636	88/1064	0·60 (0·39-0·91)	0·017	4·57	25/524	88/1064	0·64 (0·40-1·02)	0·061	4·45
Major congenital anomalies	0/636	8/1064	0 (No events)	NA	NA	0/524	8/1064	0 (No events)	NA	NA

Acronyms: ACT: artemisinin-based combination therapy, aHR: adjusted hazard ratio, AL: artemether-lumefantrine., CI: confidence interval, NA: not applicable.

* Numbers are too sparse to conduct analyses for each ACT drug, except for AL which represents >70% of all artemisinin-based treatments included in the analyses.

Adjusted by age group (<20, 20s, 30s, >=40), gravidity (1, 2, >=3) and study year (2000–4, 2005–9, 2010–17). Shared frailty Cox model was fitted to adjust for within study clustering

6-2. Subgroup analyses restricted to single exposure during the exposure risk window

Table 9. Adjusted hazard ratio of adverse pregnancy outcomes in women with confirmed exposure to artemisinin compared with women with confirmed exposure to non-artemisinin antimalarials in women with single exposure* in the embryo-sensitive period and in the first trimester

	Embryo-sensitive period (6-12 weeks gestation inclusive)					First trimester (2-13 weeks gestation inclusive)				
	ABT	non-ABT	aHR (95%CI)	p-value	E-value	ABT	non-ABT	aHR (95%CI)	p-value	E-value
Composite	37/584	54/817	0·97 (0·63-1·49)	0·892	2·57	42/735	84/1068	0·74 (0·51-1·08)	0·119	3·40
Miscarriage	23/533	42/813	1·02 (0·61-1·73)	0·932	2·72	27/668	68/1064	0·76 (0·48-1·21)	0·244	3·62
Stillbirth	12/517	6/544	1·09 (0·40-2·93)	0·870	4·45	13/641	12/655	0·65 (0·29-1·44)	0·291	6·35
Fetal loss	35/584	48/817	0·96 (0·61-1·49)	0·846	2·68	40/735	80/1068	0·70 (0·48-1·04)	0·074	3·67
Major congenital anomalies	2/584	6/817	0·86 (0·17-4·33)	0·851	11·42	2/735	4/1068	1·03 (0·19-5·73)	0·971	10·32

Acronyms: ABT: artemisinin-based treatment; aHR: adjusted hazard ratio; CI: confidence interval.
Adjusted by age group (<20, 20s, 30s, >=40), gravidity (1, 2, >=3) and study year (2000–4, 2005–9, 2010–17). Shared frailty Cox model was fitted to adjust for within study clustering

*Censored at the second exposure.

6-3. Subgroup analyses excluding non-falciparum malaria

Table 10. Adjusted hazard ratio of adverse pregnancy outcomes in women with confirmed exposure to artemisinin compared with women with confirmed exposure to non-artemisinin antimalarials in the first trimester and embryo-sensitive period excluding non-falciparum malaria cases*

	Embryo-sensitive period (6-12 weeks gestation inclusive)					First trimester (2-13 weeks gestation inclusive)				
	**ABT**	**non-ABT**	**aHR (95%CI)**	**p-value**	**E-value**	**ABT**	**non-ABT**	**aHR (95%CI)**	**p-value**	**E-value**
Composite	35/566	51/694	0·85 (0·55-1·31)	0·456	3·11	40/708	84/927	0·63 (0·43-0·93)	0·021	4·11
Miscarriage	22/515	40/690	0·89 (0·52-1·53)	0·679	3·28	26/641	68/923	0·65 (0·41-1·04)	0·075	4·35
Stillbirth	11/503	6/479	0·92 (0·34-2·52)	0·872	5·45	12/622	12/600	0·57 (0·25-1·29)	0·179	7·40
Fetal loss	33/566	46/694	0·82 (0·52-1·29)	0·389	3·31	38/708	80/927	0·60 (0·40-0·89)	0·011	4·45
Major congenital anomalies	2/566	5/694	0·93 (0·18-4·88)	0·927	10·99	2/708	4/927	0·98 (0·18-5·43)	0·979	10·96

Acronyms: ABT: artemisinin-based treatment; aHR: adjusted hazard ratio; CI: confidence interval.
Adjusted by age group (<20, 20s, 30s, >=40), gravidity (1, 2, >=3) and study year (2000–4, 2005–9, 2010–17). Shared frailty Cox model was fitted to adjust for within study clustering

*Censored at the confirmed non-falciparum malaria. Note: Malaria parasite species was only available for the SMRU data, all cases from the African sites were assumed to be *falciparum* malaria.

6-4. Subgroup analyses by narrower time-band for the exposure risk window

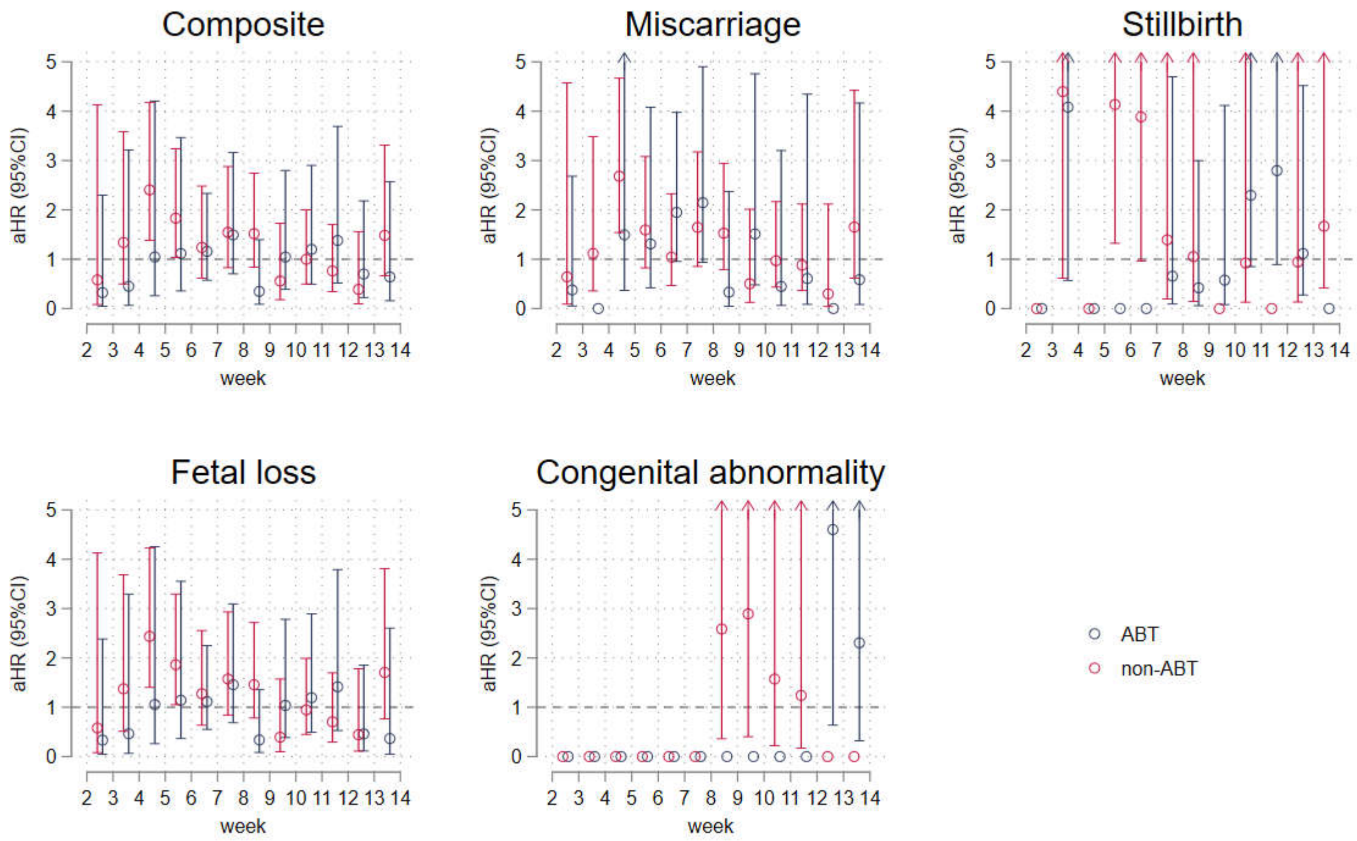

Numbers of women for the composite outcome

**week**	**2**	**3**	**4**	**5**	**6**	**7**	**8**	**9**	**10**	**11**	**12**	**13**
ABT	1/27	1/22	2/27	3/29	8/97	7/75	2/89	4/75	5/74	4/56	3/84	2/81
non-ABT	1/18	4/34	13/69	12/76	8/94	10/101	11/122	3/92	8/135	6/131	2/104	6/94

Figure 2. Adjusted hazard ratios in each exposure week for women with confirmed artemisinin-exposure (navy) or non-artemisinin-exposure (red) compared with unexposed women.

Acronyms: ABT: artemisinin-based treatment; aHR: adjusted hazard ratio; CI: confidence interval; Adjusted by age group (<20, 20s, 30s, >=40), gravidity group (1, 2, >=3) and study year (2000–4, 2005–9, 2010–17). Shared frailty Cox model was fitted to adjust for within study clustering

Women with more than one exposure are censored at the second exposure.

7. Sensitivity analyses

7-1. Different approaches to handling missing data

Table 11. Comparison of univariable, multivariable with or without multiple imputation: hazard ratio of adverse pregnancy outcomes in women with confirmed exposure to artemisinin compared with women with confirmed exposure to non- artemisinin antimalarials in the first trimester.

			Unadjusted		Adjusted* (primary model)		Adjusted with MI**		
	ABT(N)	non-ABT(N)	HR (95%CI)	p-value	aHR (95%CI)	p-value	aHR (95%CI)	p-value	E-value
Composite	42/737	96/1076	0·70 (0·49-1·02)	0·064	0·71 (0·49-1·03)	0·071	0·72 (0·50-1·05)	0·085	3·47
Miscarriage	27/670	76/1072	0·73 (0·46-1·15)	0·173	0·74 (0·47-1·17)	0·195	0·75 (0·47-1·17)	0·203	3·69
Stillbirth	13/646	12/745	0·74 (0·33-1·64)	0·454	0·71 (0·32-1·57)	0·395	0·73 (0·33-1·63)	0·445	5·58
Fetal loss	40/737	88/1076	0·69 (0·47-1·01)	0·060	0·70 (0·47-1·02)	0·065	0·71 (0·48-1·04)	0·077	3·61
Major congenital anomalies	2/737	8/1076	0·60 (0·12-2·85)	0·518	0·60 (0·13-2·87)	0·521	0·61 (0·13-2·93)	0·539	15·27

Acronyms: ABT: artemisinin-based treatment. aHR: adjusted hazard ratio. CI: confidence interval. MI: multiple imputation.
* Adjusted by age group (<20, 20s, 30s, >=40), gravidity (1, 2, >=3) and study year (2000–4, 2005–9, 2010–17). Shared frailty Cox model was fitted to adjust for within study clustering
** Adjusted by age group (<20, 20s, 30s, >=40), gravidity (1, 2, >=3), study year (2000–4, 2005–9, 2010–17), marital status (married or not), smoking status (smoker or not), previous miscarriage (yes or no) and previous stillbirth (yes or no). Shared frailty Cox model was fitted to adjust for within study clustering
Note: Age and gravidity were the only two potential confounders that were assessed across all studies. Potential confounders with missingness of less than 30% were included in the multivariable model using the multiple imputation method (Adjusted with MI model).

Table 12. Comparison of univariable, multivariable with or without multiple imputation: hazard ratio of adverse pregnancy outcomes in women with confirmed exposure to artemisinin compared with women with confirmed exposure to non-artemisinin antimalarials in the embryo-sensitive period.

			Unadjusted		Adjusted* (primary model)		Adjusted with MI**		
	ABT(N)	non-ABT(N)	HR (95%CI)	p-value	aHR (95%CI)	p-value	aHR (95%CI)	p-value	E-value
Composite	37/584	60/823	1·00 (0·66-1·53)	0·989	0·95 (0·63-1·45)	0·828	0·97 (0·64-1·48)	0·898	2·54
Miscarriage	23/533	46/819	1·09 (0·65-1·84)	0·732	1·02 (0·61-1·70)	0·951	1·02 (0·61-1·71)	0·936	2·70
Stillbirth	12/518	6/609	1·22 (0·45-3·28)	0·698	1·18 (0·44-3·18)	0·746	1·23 (0·45-3·31)	0·686	3·87
Fetal loss	35/584	52/823	1·01 (0·65-1·57)	0·959	0·96 (0·62-1·49)	0·861	0·98 (0·63-1·52)	0·932	2·58
Major congenital anomalies	2/584	8/823	0·77 (0·16-3·70)	0·743	0·72 (0·15-3·49)	0·688	0·74 (0·15-3·58)	0·711	12·56

Acronyms: ABT: artemisinin-based treatment; aHR: adjusted hazard ratio; CI: confidence interval ; MI: multiple imputation.
* Adjusted by age group (<20, 20s, 30s, >=40), gravidity (1, 2, >=3) and study year (2000–4, 2005–9, 2010–17). Shared frailty Cox model was fitted to adjust for within study clustering
** Adjusted by age group (<20, 20s, 30s, >=40), gravidity (1, 2, >=3), study year (2000–4, 2005–9, 2010–17), marital status (married or not), smoking status (smoker or not), previous miscarriage (yes or no) and previous stillbirth (yes or no). Shared frailty Cox model was fitted to adjust for within study clustering

7-2. Different approaches to handling within-cohort clustering

Table 13 Adjusted Hazard ratios of confirmed artemisinin compared with confirmed non-artemisinin in first trimester by different statistical models.

			Shared frailty (primary model)		Fixed effects		Stratified Cox	
	ABT(N)	non-ABT(N)	aHR (95%CI)	p-value	aHR (95%CI)	p-value	aHR (95%CI)	p-value
Composite	42/736	96/1074	0·71 (0·49-1·03)	0·071	0·70 (0·48-1·02)	0·060	0·72 (0·50-1·05)	0·087
Miscarriage	27/669	76/1070	0·74 (0·47-1·17)	0·195	0·73 (0·46-1·15)	0·169	0·77 (0·49-1·22)	0·265
Stillbirth	13/646	12/743	0·71 (0·32-1·57)	0·395	0·70 (0·31-1·55)	0·373	0·70 (0·32-1·56)	0·389
Fetal loss	40/736	88/1074	0·70 (0·47-1·02)	0·065	0·68 (0·46-1·00)	0·052	0·73 (0·50-1·08)	0·116
Major congenital anomalies	2/736	8/1074	0·60 (0·13-2·87)	0·521	0·63 (0·13-3·00)	0·560	0·63 (0·13-3·00)	0·558

Acronyms: ABT: artemisinin-based treatment; aHR: adjusted hazard ratio; CI: confidence interval.
All models are adjusted by age group (<20, 20s, 30s, >=40), gravidity (1, 2, >=3) and study year (2000–4, 2005–9, 2010–17).
Within-study clustering is adjusted either as shared frailty, as a covariate (fixed-effects) or by stratification (stratified Cox).
Shared frailty is the main model presented in Figure 2 in the main manuscript.

7-3. Analyses excluding confirmed HIV-positive women

Table 14. Adjusted hazard ratio of adverse pregnancy outcomes in women with confirmed exposure to artemisinin compared with women with confirmed exposure to non-artemisinin antimalarials excluding HIV-confirmed women.

	Embryo-sensitive period (6-12 weeks gestation inclusive)					First trimester (2-13 weeks gestation inclusive)				
	ABT	non-ABT	aHR (95%CI)	p-value	E-value	ABT	non-ABT	aHR (95%CI)	p-value	E-value
Composite	36/557	60/818	0·98 (0·64-1·50)	0·934	2·51	40/700	96/1069	0·72 (0·49-1·05)	0·089	3·51
Miscarriage	23/510	46/814	1·08 (0·64-1·81)	0·768	2·51	26/638	76/1065	0·76 (0·48-1·21)	0·251	3·60
Stillbirth	11/492	6/604	1·14 (0·42-3·12)	0·800	4·29	12/612	12/738	0·69 (0·31-1·56)	0·378	6·03
Fetal loss	34/557	52/818	0·99 (0·63-1·54)	0·958	2·56	38/700	88/1069	0·70 (0·48-1·04)	0·079	3·66
Major congenital anomalies	2/557	8/818	0·74 (0·15-3·54)	0·701	12·71	2/700	8/1069	0·61 (0·13-2·91)	0·534	15·39

Acronyms: ABT: artemisinin-based treatment; aHR: adjusted hazard ratio; CI: confidence interval; MI: multiple imputation.

Adjusted by age group (<20, 20s, 30s, >=40), gravidity (1, 2, >=3) and study year (2000–4, 2005–9, 2010–17). Shared frailty Cox model was fitted to adjust for within study clustering

*Censored at the confirmed non-falciparum malaria. Note: Malaria parasite species was only available for the SMRU data, all cases from the African sites were assumed to be falciparum malaria.

7-4. Assessment of study-specific data and influence of individual cohorts

Table 15. Number of confirmed antimalarial exposures included in the final analysis and crude number of exposures in the original dataset in each study cohort.

Cohort	**All**	**Unexposed**	**ABT**	**ACT**	**AL**	**ASAQ**	**ASMQ**	**DP**	**Non-ABT**	**Oral quinine**
Rouamba, Burkina Faso	4519/5187	4354/4652	13/44	11/41	6/9	3/21	0/0	3/11	152/501	147/322
WHO TDR, multi country*	855/1158	834/935	11/74	10/60	8/44	1/10	0/0	2/6	10/158	4/58
Tinto, Burkina Faso	677/714	626/652	30/40	30/40	1/1	29/39	0/0	0/0	21/22	21/22
Dellicour, Kenya	1148/1453	1075/1084	71/341	71/328	71/328	0/0	0/0	0/0	2/39	2/34
Mosha, Tanzania	1752/1783	1527/1549	156/165	156/165	156/165	0/0	0/0	0/0	69/69	69/69
Sevene, Mozambique	734/763	710/733	19/23	19/23	19/23	0/0	0/0	0/0	5/5	5/5
Rulisa, Rwanda	1648/2070	1571/1992	77/78	77/78	77/78	0/0	0/0	0/0	0/0	0/0
Manyando, Zambia	935/1001	763/819	166/176	166/173	166/173	0/0	0/0	0/0	6/6	6/9
McGready, Thailand-Myanmar	21910/24867	20905/23812	194/347	98/120	26/27	0/0	62/63	24/26	811/841	714/771
Total	34178/38996	32365/36228	737/1288	638/1028	530/848	33/70	62/63	29/43	1076/1641	968/1290

Acronyms: ABT: artemisinin-based treatment; ACT: artemisinin-based combination therapies; AL: artemether-lumefantrine; ASAQ: artesunate-amodiaquine; ASMQ: artesunate-mefloquine ; DP: dihydroartemisinin-piperaquine.

The numerator is the number of exposures included in the final analysis after applying exclusion criteria. These numbers include women who were exposed to the same class antimalarials twice or more, so the numbers in subgroup analyses can be smaller than the numbers in this table.

The denominator is the number of confirmed and unconfirmed exposures before applying the inclusion criteria (Excluding women enrolled at or after delivery, if the estimated gestational age was missing, the fetus was confirmed unviable at enrolment, or exposure information was incomplete. Multiple gestation pregnancies (e.g. twins) were also excluded as described in the methods).

Table 16. Prevalence of the composite outcome in each study cohort by exposure groups.

Cohort	Unexposed	ABT First trimester	Non-ABT First trimester	ABT Embryo-sensitive period	Non-ABT Embryo-sensitive period
Rouamba, Burkina Faso	92/4354	0/13	2/152	0/9	2/119
WHO TDR, Ghana	9/246	0/5	0/4	0/3	0/3
WHO TDR, Kenya	0/230	0/3	0/3	0/2	0/3
WHO TDR, Tanzania	13/187	0	0	0	0
WHO TDR, Uganda	2/171	0/3	0/3	0/3	0/3
Tinto, Burkina Faso	18/626	0/30	1/21	0/21	1/13
Dellicour, Kenya	90/1075	3/71	0/2	1/47	0/1
Mosha, Tanzania	82/1527	10/156	7/69	10/154	3/33
Sevene, Mozambique	32/710	0/19	0/5	0/20	0/4
Rulisa, Rwanda	59/1571	3/77	0	3/71	0
Manyando, Zambia	17/763	8/166	1/6	8/132	1/6
McGready, Thailand-Myanmar	1979/20905	18/194	85/811	15/122	53/638

ABT: artemisinin-based treatment

Note: The total number of events and pregnancies do not always add up to the same number between two separate risk periods because women were categorized by the first antimalarial exposure and were censored at the time of exposure to an antimalarial of another exposure group (i.e. ABT or non-ABT). The exposures were assessed separately for each risk period (first trimester and embryo-sensitive period), and exposures outside each risk period were not considered.

Table 17. Prevalence of the miscarriage in each study cohort by exposure groups.

Cohort	Unexposed	ABT First trimester	Non-ABT First trimester	ABT Embryo-sensitive period	Non-ABT Embryo-sensitive period
Rouamba, Burkina Faso	11/4130	0/13	0/151	0/9	0/118
WHO TDR, Ghana	5/236	0/5	0/4	0/3	0/3
WHO TDR, Kenya	0/167	0/3	0/3	0/2	0/3
WHOTDR, Tanzania	7/176	0	0	0	0
WHO TDR, Uganda	1/88	0/3	0/1	0/3	0/1
Tinto, Burkina Faso	5/437	0/23	1/21	0/17	1/13
Dellicour, Kenya	60/905	3/62	0/2	1/41	0/1
Mosha, Tanzania	29/1527	4/156	3/69	4/154	1/33
Sevene, Mozambique	12/608	0/15	0/5	0/16	0/4
Rulisa, Rwanda	12/674	1/61	0	1/58	0
Manyando, Zambia	3/447	6/135	0/5	6/108	0/5
McGready, Thailand-Myanmar	1662/20905	13/194	72/811	11/122	44/638

ABT: artemisinin-based treatment

Note: The total number of events and pregnancies do not always add up to the same number between two separate risk periods because women were categorized by the first antimalarial exposure and were censored at the time of exposure to an antimalarial of another exposure group (i.e. ABT or non-ABT). The exposures were assessed separately for each risk period (first trimester and embryo-sensitive period), and exposures outside each risk period were not considered.

Table 18. Prevalence of fetal loss in each study cohort by exposure groups.

Cohort	Unexposed	ABT First trimester	Non-ABT First trimester	ABT Embryo-sensitive period	Non-ABT Embryo-sensitive period
Rouamba, Burkina Faso	88/4354	0/13	2/152	0/9	2/119
WHO TDR, Ghana	9/246	0/5	0/4	0/3	0/3
WHO TDR, Kenya	0/230	0/3	0/3	0/2	0/3
WHOTDR, Tanzania	13/187	0	0	0	0
WHO TDR, Uganda	2/171	0/3	0/3	0/3	0/3
Tinto, Burkina Faso	18/626	0/30	1/21	0/21	1/13
Dellicour, Kenya	84/1075	3/71	0/2	1/47	0/1
Mosha, Tanzania	80/1527	10/156	7/69	10/154	3/33
Sevene, Mozambique	30/710	0/19	0/5	0/20	0/4
Rulisa, Rwanda	58/1571	3/77	0	3/71	0
Manyando, Zambia	17/763	8/166	1/6	8/132	1/6
McGready, Thailand-Myanmar	1812/20905	16/194	77/811	13/122	45/638

ABT: artemisinin-based treatment

Note: The total number of events and pregnancies do not always add up to the same number between two separate risk periods because women were categorized by the first antimalarial exposure and were censored at the time of exposure to an antimalarial of another exposure group (i.e. ABT or non-ABT). The exposures were assessed separately for each risk period (first trimester and embryo-sensitive period), and exposures outside each risk period were not considered.

Table 19. Prevalence of major congenital anomalies among live-births in each study cohort by exposure groups.

Cohort	Unexposed	ABT First trimester	Non-ABT First trimester	ABT Embryo-sensitive period	Non-ABT Embryo-sensitive period
Rouamba, Burkina Faso	4/3933 (0·1%)	0/10	0/137	0/7	0/105
WHO TDR, Ghana	0/202	0/4	0/3	0/3	0/2
WHO TDR, Kenya	0/224	0/3	0/3	0/2	0/3
WHO TDR, Tanzania	0/136	0/0	0/0	0/0	0/0
WHO TDR, Uganda	0/152	0/1	0/3	0/1	0/3
Tinto, Burkina Faso	0/608	0/29	0/20	0/21	0/12
Dellicour, Kenya	6/804 (0·8%)	0/59	0/2	0/38	0/1
Mosha, Tanzania	2/1444 (0·1%)	0/146	0/62	0/144	0/30
Sevene, Mozambique	2/659 (0·3%)	0/19	0/4	0/20	0/4
Rulisa, Rwanda	1/1513 (0·08%)	0/74	0/0	0/68	0/0
Manyando, Zambia	0/621	0/158	0/5	0/124	0/5
McGready, Thailand-Myanmar	167/15974 (1·1%)	2/120 (1·7%)	8/442 (1·8%)	2/75 (2·7%)	8/393 (2·0%)

ABT: artemisinin-based treatment
Congenital heart defects were reported on the Thailand-Myanmar border (12 in unexposed and 1 in Non-ABT-exposed), Kenya (2 in unexposed) and Tanzania (1 in unexposed).

Note: The total number of events and pregnancies do not always add up to the same number between two separate risk periods because women were categorized by the first antimalarial exposure and were censored at the time of exposure to an antimalarial of another exposure group (i.e. ABT or non-ABT). The exposures were assessed separately for each risk period (first trimester and embryo-sensitive period), and exposures outside each risk period were not considered.

Table 20. Variable missingness in each study cohort.

	Rouamba, Burkina Faso	WHO TDR, Ghana	WHO TDR, Kenya	WHO TDR, Tanzania	WHO TDR, Uganda	Tinto, Burkina Faso
Age	0·5% (21/4519)	0·4% (1/255)	0% (0/236)	0% (0/187)	0% (0/177)	0% (0/677)
Gravidity	0·1% (5/4519)	0·4% (1/255)	0·4% (1/236)	2·1% (4/187)	0% (0/177)	0% (0/677)
Parity	0·2% (8/4519)	0·4% (1/255)	0·8% (2/236)	2·1% (4/187)	0% (0/177)	0% (0/677)
Previous miscarriage	43·3% (1958/4519)	1·2% (3/255)	2·1% (5/236)	1·1% (2/187)	2·3% (4/177)	0% (0/677)
Previous stillbirth	43·4% (1961/4519)	1·2% (3/255)	2·1% (5/236)	1·6% (3/187)	2·3% (4/177)	0% (0/677)
Height	100% (4519/4519)	100% (255/255)	100% (236/236)	100% (187/187)	100% (177/177)	100% (677/677)
Body weight	100% (4519/4519)	100% (255/255)	100% (236/236)	100% (187/187)	100% (177/177)	100% (677/677)
Marital status: married	100% (4519/4519)	100% (255/255)	100% (236/236)	100% (187/187)	100% (177/177)	0% (0/677)
HIV	64·9% (2932/4519)	69·8% (178/255)	50·4% (119/236)	10·2% (19/187)	76·3% (135/177)	3% (20/677)
Smoking	100% (4519/4519)	100% (255/255)	100% (236/236)	100% (187/187)	100% (177/177)	0% (0/677)
Alcohol	100% (4519/4519)	100% (255/255)	100% (236/236)	100% (187/187)	100% (177/177)	0% (0/677)
Literacy	100% (4519/4519)	100% (255/255)	100% (236/236)	100% (187/187)	100% (177/177)	100% (677/677)
Education	100% (4519/4519)	100% (255/255)	100% (236/236)	100% (187/187)	100% (177/177)	0% (0/677)
IPTp doses	0% (0/4519)	100% (255/255)	100% (236/236)	100% (187/187)	0% (0/177)	0% (0/677)
Gestational age estimation method	5·8% (260/4519)	25·1% (64/255)	3·4% (8/236)	45·5% (85/187)	13·6% (24/177)	0% (0/677)

Table 20 continued

	Dellicour, Kenya	Mosha, Tanzania	Sevene, Mozambique	Rulisa, Rwanda	Manyando, Zambia	McGready, Thailand-Myanmar
Age	0% (0/1148)	0% (0/1752)	0·3% (2/734)	4·4% (72/1648)	0% (0/935)	0% (1/21910)
Gravidity	4·3% (49/1148)	0·5% (8/1752)	0·3% (2/734)	4·4% (72/1648)	0% (0/935)	0% (0/21910)
Parity	100% (1148/1148)	100% (1752/1752)	0% (0/734)	100% (1648/1648)	100% (935/935)	0% (0/21910)
Previous miscarriage	6% (69/1148)	50·2% (880/1752)	0·3% (2/734)	54·7% (901/1648)	51·9% (485/935)	0% (0/21910)
Previous stillbirth	6·4% (74/1148)	50·2% (880/1752)	0% (0/734)	54·7% (901/1648)	51·9% (485/935)	2·8% (621/21910)
Height	32·7% (375/1148)	100% (1752/1752)	0·3% (2/734)	100% (1648/1648)	100% (935/935)	100% (21910/21910)
Body weight	31·2% (358/1148)	100% (1752/1752)	0·3% (2/734)	100% (1648/1648)	100% (935/935)	95·8% (20987/21910)
Marital status: married	4·1% (47/1148)	2·2% (38/1752)	0·1% (1/734)	100% (1648/1648)	100% (935/935)	0% (0/21910)
HIV	11·3% (130/1148)	5·5% (96/1752)	9·5% (70/734)	5·3% (87/1648)	0·3% (3/935)	100% (21910/21910)
Smoking	30·9% (355/1148)	100% (1752/1752)	0·5% (4/734)	100% (1648/1648)	100% (935/935)	0·4% (83/21910)
Alcohol	30·9% (355/1148)	100% (1752/1752)	0·7% (5/734)	100% (1648/1648)	100% (935/935)	100% (21910/21910)
Literacy	100% (1148/1148)	100% (1752/1752)	100% (734/734)	100% (1648/1648)	100% (935/935)	60·5% (13265/21910)
Education	4·4% (51/1148)	1% (18/1752)	0·3% (2/734)	100% (1648/1648)	0·1% (1/935)	100% (21910/21910)
IPTp doses	0% (0/1148)	100% (1752/1752)	4·8% (35/734)	100% (1648/1648)	100% (935/935)	NA
Gestational age estimation method	0% (0/1148)	100% (1752/1752)	1·2% (9/734)	100% (1648/1648)	0% (0/935)	0% (0/21910)

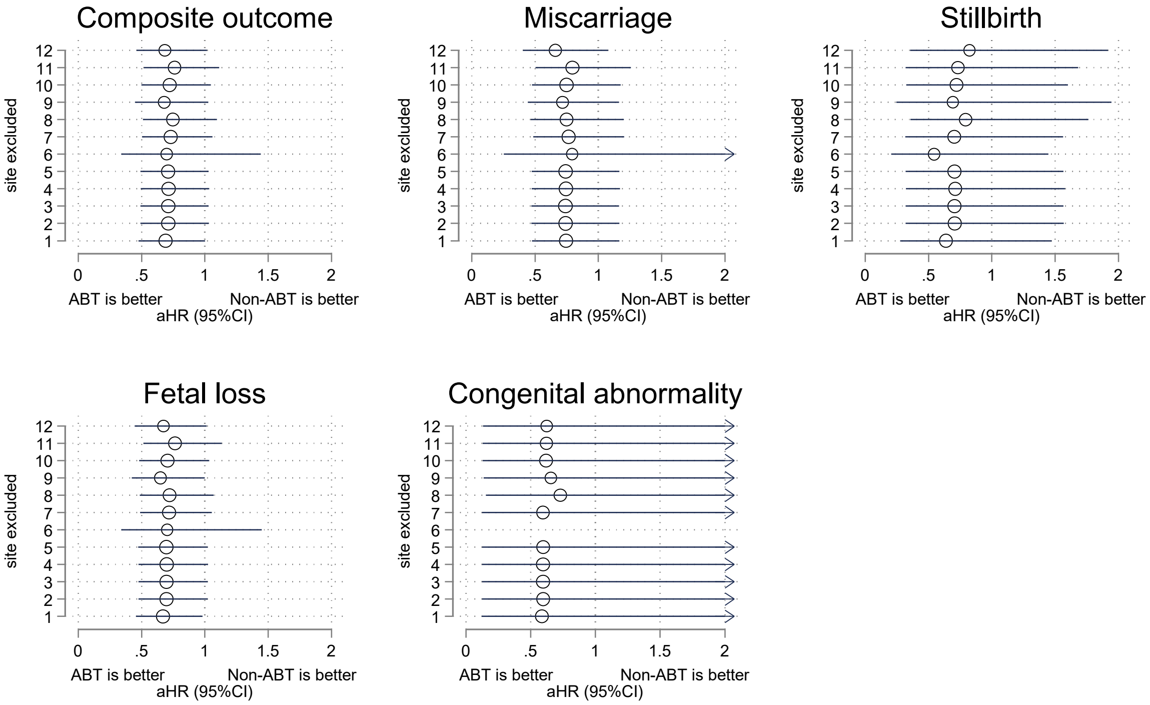

Figure 3. Sensitivity analysis excluding one cohort at a time to identify any influential cohorts: hazard ratio comparing confirmed exposure, artemisinin-based treatment (ABT) and non-artemisinin treatments (non-ABT) in the first trimester.

Study cohort legend:

1: Rouamba, Burkina Faso;

2: WHO TDR, Ghana;

3: WHO TDR, Kenya;

4: WHO TDR, Tanzania;

5: WHO TDR, Uganda;

6: McGready, Thailand-Myanmar;

7: Tinto, Burkina Faso;

8: Dellicour, Kenya;

9: Mosha, Tanzania;

10: Sevene, Mozambique;

11: Rulisa, Rwanda;

12: Manyando, Zambia.

Acronyms: ABT: artemisinin-based treatment. aHR: adjusted hazard ratio. CI: confidence interval.

The size of the circle is proportional to the number of artemisinin-exposed women included.

There was no congenital abnormality when site 6 was excluded.

Hazard ratios are adjusted by age group (<20, 20s, 30s, >=40), gravidity (1, 2, >=3) and study year (2000–4, 2005–9, 2010–17). Shared frailty Cox model was fitted to adjust for within study clustering

The results excluding site 6 are the pooled results for sub-Saharan Africa.

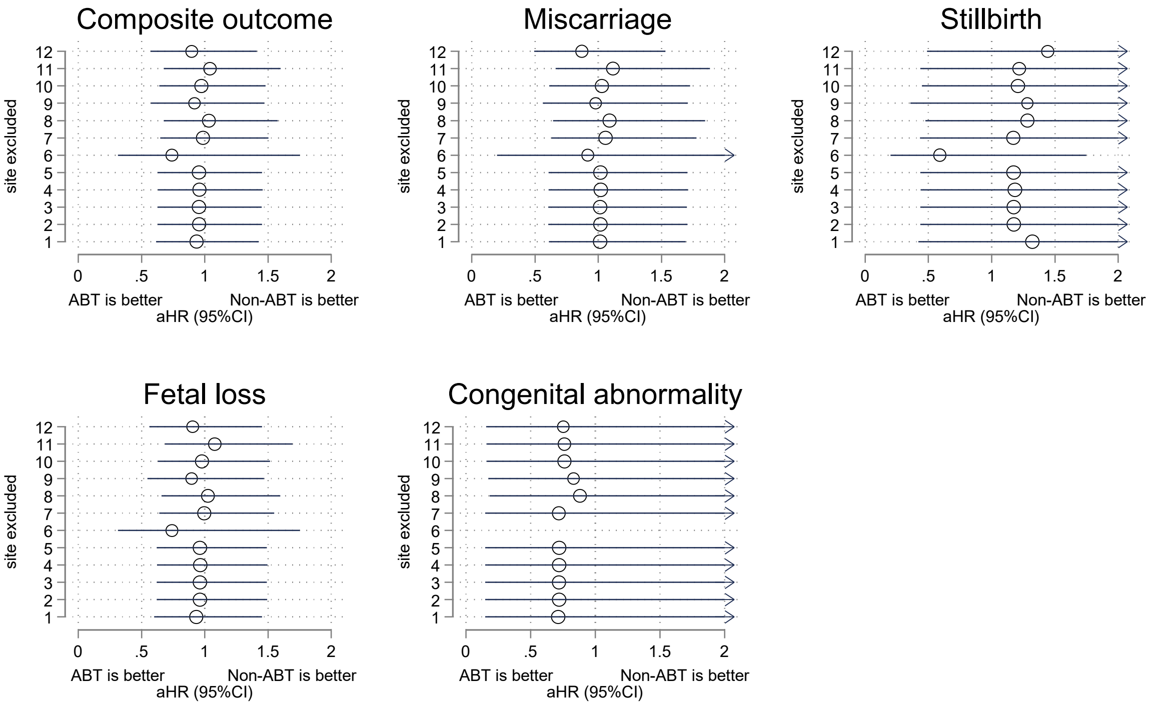

Figure 4. Sensitivity analysis excluding one cohort at a time to identify any influential cohorts: hazard ratio comparing confirmed exposure, artemisinin-based treatment (ABT) and non-artemisinin treatments (non-ABT) in the embryo-sensitive period.

Study cohort legend:

1: Rouamba, Burkina Faso;

2: WHO TDR, Ghana;

3: WHO TDR, Kenya;

4: WHO TDR, Tanzania;

5: WHO TDR, Uganda;

6: McGready, Thailand-Myanmar;

7: Tinto, Burkina Faso;

8: Dellicour, Kenya;

9: Mosha, Tanzania;

10: Sevene, Mozambique;

11: Rulisa, Rwanda;

12: Manyando, Zambia.

Acronym: ABT: artemisinin-based treatment. aHR: adjusted hazard ratio. CI: confidence interval.
The size of the circle is proportional to the number of artemisinin-exposed women included.
There was no congenital abnormality when site 6 was excluded.
Hazard ratios are adjusted by age group (<20, 20s, 30s, >=40), gravidity group (1, 2, >=3) and study year (2000–4, 2005–9, 2010–17). Shared frailty Cox model was fitted to adjust for within study clustering
The results excluding site 6 are the pooled results for sub-Saharan Africa.

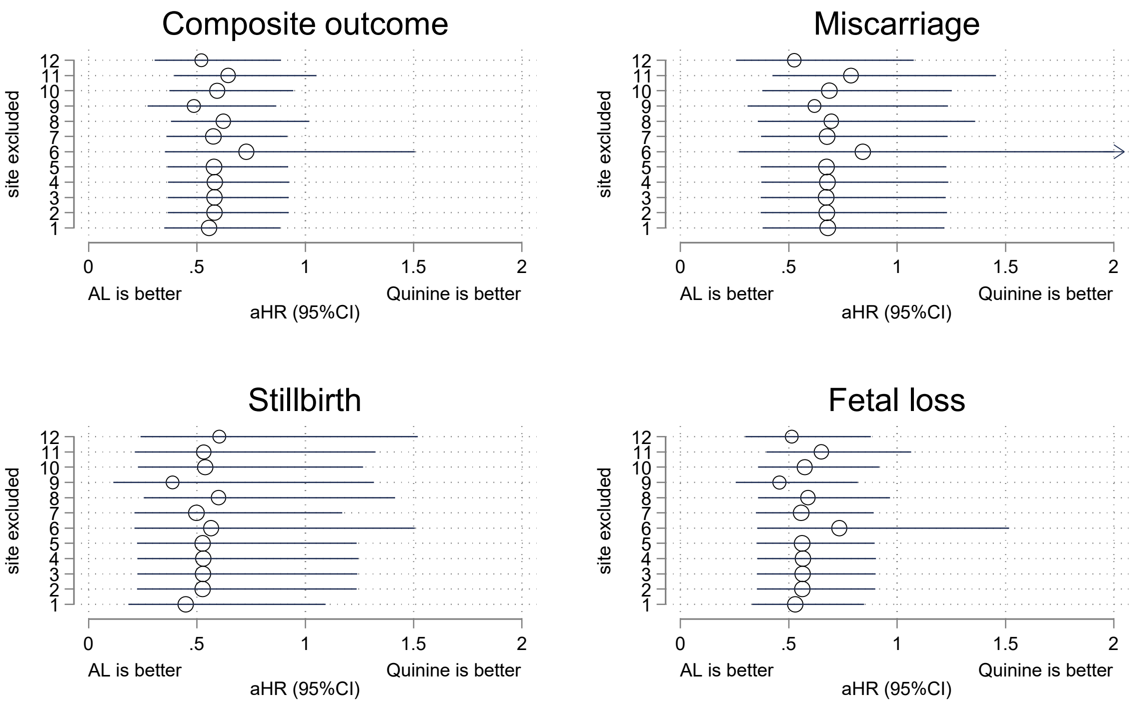

Figure 5. Sensitivity analysis excluding one cohort at a time to identify any influential cohorts: hazard ratio comparing confirmed artemether-lumefantrine compared with oral quinine in the first trimester.

Study cohort legend:

1: Rouamba, Burkina Faso;
2: WHO TDR, Ghana;
3: WHO TDR, Kenya;
4: WHO TDR, Tanzania;
5: WHO TDR, Uganda;
6: McGready, Thailand-Myanmar;
7: Tinto, Burkina Faso;
8: Dellicour, Kenya;
9: Mosha, Tanzania;
10: Sevene, Mozambique;
11: Rulisa, Rwanda;
12: Manyando, Zambia.

Acronyms: aHR: adjusted hazard ratio. AL: artemether-lumefantrine. CI: confidence interval.

The size of the circle is proportional to the number of artemisinin-exposed women included. There was no congenital abnormality in women exposed to artemether-lumefantrine.

Hazard ratios are adjusted by age group (<20, 20s, 30s, >=40), gravidity (1, 2, >=3), and study year (2000–4, 2005–9, 2010–17). Shared frailty Cox model was fitted to adjust for within study clustering

The results excluding site 6 are the pooled results for sub-Saharan Africa.

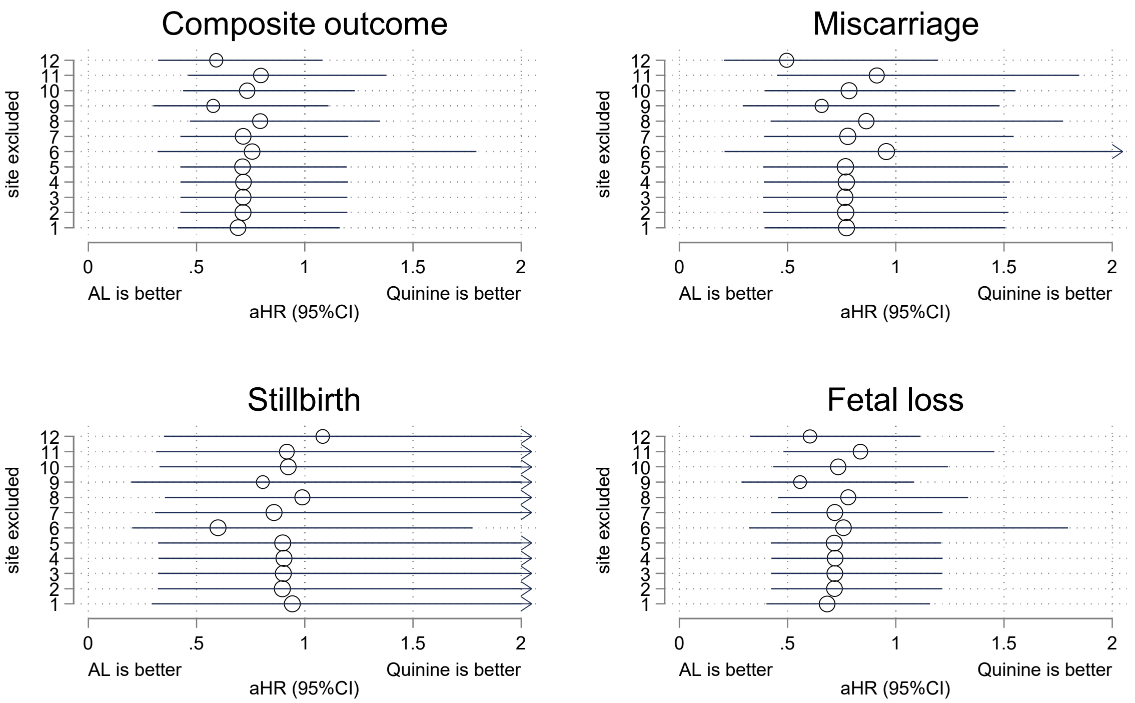

Figure 6. Sensitivity analysis excluding one cohort at a time to identify any influential cohorts: hazard ratio comparing confirmed artemether-lumefantrine compared with oral quinine in embryo-sensitive period.

<u>Study cohort legend:</u>

1: Rouamba, Burkina Faso;

2: WHO TDR, Ghana;

3: WHO TDR, Kenya;

4: WHO TDR, Tanzania;

5: WHO TDR, Uganda;

6: McGready, Thailand-Myanmar;

7: Tinto, Burkina Faso;

8: Dellicour, Kenya;

9: Mosha, Tanzania;

10: Sevene, Mozambique;

11: Rulisa, Rwanda;

12: Manyando, Zambia.

Acronyms: aHR: adjusted hazard ratio. AL: artemether-lumefantrine. CI: confidence interval.

The size of the circle is proportional to the number of artemisinin-exposed women included. There was no congenital abnormality in women exposed to artemether-lumefantrine.

Hazard ratios are adjusted by age group (<20, 20s, 30s, >=40), gravidity group (1, 2, >=3) and study year (2000–4, 2005–9, 2010–17). Shared frailty Cox model was fitted to adjust for within study clustering

The results excluding site 6 are the pooled results for sub-Saharan Africa.

7-5. Analyses including confirmed and unconfirmed exposures

Table 21. Characteristics of women with confirmed or unconfirmed exposure to artemisinin or non-artemisinin in the embryo-sensitive period and those unexposed in the embryo-sensitive period

	Unexposed (n=32312)		Artemisinin-exposed (n=1024)*		Non-artemisinin-exposed (n=1235)**	
	N	mean(SD)/% (n)	N	mean(SD)/% (n)	N	mean(SD)/% (n)
EGA at enrolment	32312	12·9 (7·3)	1024	15·7 (8·5)	1235	10·5 (5·4)
Duration of follow-up	32312	22·4 (9·9)	1024	20·6 (9·6)	1235	22·8 (10·1)
EGA at exposure		NA	1024	8·5 (3·1)	1235	8·8 (3)
Pregnancy outcome available	32312	88·8 (28698)	1024	93·1 (953)	1235	73·6 (909)
**Baseline characteristics**						
Age (years)	32215	26.1 (6.5)	1024	25.9 (6.5)	1233	25.2 (6.5)
Gravidity	32173		1023		1232	
1		29·3 (9440)		37·0 (378)		30·4 (374)
2		20·8 (6684)		16·4 (168)		20·2 (249)
≥3		49·9 (16049)		46·6 (477)		49·4 (609)
Parity	27411		323		1142	
0		29·8 (8173)		36·8 (119)		34·3 (392)
1		22·8 (6258)		22·6 (73)		20·8 (238)
≥2		47·4 (12980)		40·6 (131)		44·8 (512)
Previous miscarriage (Yes)	28246	21·5 (6073)	827	14·0 (116)	1188	25·7 (305)
Previous stillbirth (Yes)	27676	2·7 (759)	821	2·3 (19)	1136	3·5 (40)
Height (m)	1384	1·6 (0·1)	257	1·6 (0·1)	18	1·6 (0·1)
Body weight (kg)	1408	59·8 (9·6)	410	54·2 (10·1)	783	46·5 (6·9)
Marital status: married	24717	96·9 (23939)	695	85·3 (593)	920	98·7 (908)
HIV (positive)	7731	8·2 (632)	741	12·6 (93)	298	5·4 (16)
Smoking: yes	22862	21 (4805)	484	10·5 (51)	838	36·4 (305)
Alcohol: yes	2025	14·6 (295)	292	5·5 (16)	39	23·1 (9)
Literate	8467	62·6 (5300)	65	46·2 (30)	113	45·1 (51)
Education	4585		671		115	
No		19·3 (886)		24·1 (162)		8·7 (10)
primary		61·5 (2821)		55·0 (369)		71·3 (82)
secondary or higher		19·1 (878)		20·9 (140)		20·0 (23)
IPTp doses	6850		399		314	
0		21·6 (1479)		38·1 (152)		17·2 (54)
1		32·0 (2193)		19·8 (79)		31·8 (100)
2		39·2 (2684)		18·8 (75)		44·3 (139)
3		5·6 (383)		16·0 (64)		5·7 (18)
4		1·6 (111)		7·3 (29)		1·0 (3)
N/A	20905		194		811	
Gestational age by ultrasound	28780	69·7 (20050)	780	42·9 (335)	1139	54·9 (625)
Country	32312		1024		1235	
Burkina Faso		15·4 (4979)		5·6 (57)		22·8 (281)
Ghana		0·8 (245)		0·9 (9)		0·3 (4)
Kenya		3·9 (1255)		31·6 (324)		2·1 (26)
Tanzania		5·3 (1714)		15·2 (156)		7·2 (89)

	Unexposed (n=32312)		Artemisinin-exposed (n=1024)*		Non-artemisinin-exposed (n=1235)**	
	**N**	**mean(SD)/% (n)**	**N**	**mean(SD)/% (n)**	**N**	**mean(SD)/% (n)**
Uganda		0·5 (170)		1·9 (19)		1·1 (13)
Mozambique		2·2 (710)		2·1 (22)		0·4 (5)
Rwanda		4·9 (1571)		7·5 (77)		0 (0)
Zambia		2·4 (763)		16·2 (166)		0·5 (6)
Thailand-Myanmar		64·7 (20905)		18·9 (194)		65·7 (811)
Study year	32312		1024		1235	
2000–2004		16·2 (5243)		5·4 (55)		30·3 (374)
2005–2009		30·9 (9986)		31·1 (318)		29·5 (364)
2010–2017		52·9 (17083)		63·6 (651)		40·2 (497)

Acronyms: EGA: estimated gestational age; IPTp: intermittent preventive treatment in pregnancy; N: number of women evaluated; N/A: not applicable; SD: standard deviation.

Women are categorised according to the first exposure in the first trimester.

* including 917 ACT (788 AL, 42 ASAQ, 58 ASMQ, 26 DP, 3 artesunate-atovaquone-proguanil), 95 AS/AC, 8 parenteral, 3 artemether based on the first exposure in the first trimester

** including 1043 oral quinine (841 quinine monotherapy, 202 quinine+clindamycin), 32 parenteral quinine, 147 chloroquine, 8 amodiaquine,1 atovaquone-proguanil, 1 mefloquine, 1 quinine+mefloquine, 2 details not available, based on the first exposure in the first trimester.

Table 22. Number of exposure and outcomes for each artemisinin-based treatment in the first trimester and embryo-sensitive period regardless of confirmation of exposure

Outcome	ABT	ACT	AL	ASAQ	ASMQ	DP	AS/AC
**First trimester (regardless of confirmation)**							
Composite outcome	69/1024	57/918	57/788	0/42	5/58	1/27	10/97
Miscarriage	52/915	44/810	44/688	0/35	4/58	1/26	7/97
Stillbirth	15/907	13/836	13/728	0/41	1/42	0/22	1/63
Fetal loss	67/1024	57/918	57/788	0/42	5/58	1/27	8/97
Major congenital abnormality	2/1024	0/918	0/788	0/42	0/58	0/27	2/97
**Embryo-sensitive period (regardless of confirmation)**							
Composite outcome	54/770	46/695	46/617	0/29	6/29	1/18	8/71
Miscarriage	39/690	33/616	33/542	0/25	4/29	1/18	6/71
Stillbirth	13/687	12/639	12/571	0/28	1/22	0/16	1/44
Fetal loss	52/770	45/695	45/617	0/29	5/29	1/18	7/71
Major congenital abnormality	2/770	1/695	1/617	0/29	1/29	0/18	1/71

Women who were exposed to different ACTs are counted one for each group and may not add up to ABT due to cases with multiple treatment

Acronyms: ABT: artemisinin-based treatment; ACT: artemisinin combination therapies; AL: artemether-lumefantrine ; ASAQ: artesunate-amodiaquine ; ASMQ: artesunate-mefloquine ; DP: dihydroartemisinin-piperaquine ; AS/AC: artesunate monotherapy/artesunate-clindamycin.

Table 23. Adjusted hazard ratio of adverse pregnancy outcomes in women with exposure to artemisinin compared with women with exposure to non-artemisinin antimalarials regardless of the confirmation of the exposures.

	Embryo-sensitive period (6-12 weeks gestation inclusive)					First trimester (2-13 weeks gestation inclusive)				
	**ABT**	**non-ABT**	**aHR (95%CI)**	**p-value**	**E-value**	**ABT**	**non-ABT**	**aHR (95%CI)**	**p-value**	**E-value**
Composite	54/770	64/915	1·06 (0·73-1·54)	0·753	2·11	69/1023	100/1230	0·84 (0·61-1·16)	0·283	2·70
Miscarriage	39/690	47/899	1·29 (0·83-2·01)	0·251	1·72	52/914	77/1211	0·97 (0·67-1·41)	0·872	2·39
Stillbirth	13/687	9/697	0·80 (0·34-1·91)	0·622	5·40	15/907	15/892	0·61 (0·29-1·27)	0·184	6·38
Fetal loss	52/770	56/915	1·08 (0·73-1·59)	0·711	2·11	67/1023	92/1230	0·83 (0·60-1·15)	0·265	2·77
Major congenital anomalies	2/770	8/915	0·59 (0·12-2·85)	0·509	16·20	2/1023	8/1230	0·47 (0·10-2·28)	0·348	20·42

Acronyms: ABT: artemisinin-based treatment; aHR: adjusted hazard ratio; CI: confidence interval.

Adjusted by age group (<20, 20s, 30s, >=40), gravidity (1, 2, >=3) and study year (2000–4, 2005–9, 2010–17). Shared frailty Cox model was fitted to adjust for within study clustering

Table 24. Hazard ratio of adverse pregnancy outcomes in women with exposure (confirmed and unconfirmed) to artemisinin or non-artemisinin antimalarials compared with women unexposed to antimalarials in the embryo-sensitive period and the first trimester

	Embryo-sensitive period (6-12 wks gestation inclusive)				First trimester (2-13 weeks gestation inclusive)			
	**Exposed**	**Unexposed**	**aHR (95% CI)**	**p-value**	**Exposed**	**Unexposed**	**aHR (95% CI)**	**p-value**
**Composite**								
ABT	54/770	2454/32735	1·17 (0·88-1·55)	0·276	69/1023	2393/32150	1·07 (0·83-1·38)	0·606
Non-ABT	64/915		1·10 (0·86-1·41)	0·448	100/1230		1·27 (1·04-1·56)	0·019
**Miscarriage**								
ABT	39/690	1859/30675	1·39 (0·99-1·95)	0·055	52/914	1807/30123	1·25 (0·93-1·68)	0·147
Non-ABT	47/899		1·07 (0·80-1·44)	0·633	77/1211		1·28 (1·02-1·62)	0·033
**Stillbirth**								
ABT	13/687	412/28325	0·78 (0·44-1·37)	0·381	15/907	404/27847	0·71 (0·42-1·21)	0·207
Non-ABT	9/697		0·97 (0·50-1·87)	0·917	15/892		1·17 (0·70-1·96)	0·557
**Fetal loss**								
ABT	52/770	2271/32735	1·11 (0·84-1·48)	0·463	67/1023	2211/32150	1·03 (0·80-1·33)	0·815
Non-ABT	56/915		1·03 (0·79-1·35)	0·802	92/1230		1·24 (1·01-1·53)	0·043
**Major congenital anomalies**								
ABT	2/770	183/32735	1·09 (0·26-4·53)	0·904	2/1023	182/32150	0·74 (0·18-3·08)	0·680
Non-ABT	8/915		1·86 (0·91-3·80)	0·089	8/1230		1·58 (0·77-3·23)	0·209

Acronyms: ABT: artemisinin-based treatment. aHR: adjusted hazard ratio. CI: confidence interval.

Adjusted by age group (<20, 20s, 30s, >=40), gravidity group (1, 2, >=3) and study year (2000–4, 2005–9, 2010–17). Shared frailty Cox model was fitted to adjust for within study clustering

Note: the hazard ratios compared ABT-exposed women with women unexposed to any antimalarials in the risk period are shown. These are derived from the same model as the results shown in **Table 23**: only the reference group is different.

8. Supplemental references

1. Dellicour S, Sevene E, McGready R, et al. First-trimester artemisinin derivatives and quinine treatments and the risk of adverse pregnancy outcomes in Africa and Asia: A meta-analysis of observational studies. *PLoS Med* 2017; **14**(5): e1002290.
2. Mehta U, Clerk C, Allen E, et al. Protocol for a drugs exposure pregnancy registry for implementation in resource-limited settings. *BMC Pregnancy Childbirth* 2012; **12**: 89.
3. Rouamba T, Valea I, Bognini JD, et al. Safety Profile of Drug Use During Pregnancy at Peripheral Health Centres in Burkina Faso: A Prospective Observational Cohort Study. *Drugs Real World Outcomes* 2018; **5**(3): 193-206.
4. Tinto H, Sevene E, Dellicour S, et al. Assessment of the safety of antimalarial drug use during early pregnancy (ASAP): protocol for a multicenter prospective cohort study in Burkina Faso, Kenya and Mozambique. *Reproductive health* 2015; **12**: 112.
5. Matteo Q, Simon G, James C. jomo: A Flexible Package for Two-level Joint Modelling Multiple Imputation. *R J* 2019; **11**(2): 205-28.
6. Manyando C, Mkandawire R, Puma L, et al. Safety of artemether-lumefantrine in pregnant women with malaria: results of a prospective cohort study in Zambia. *Malar J* 2010; **9**: 249.
7. Rulisa S, Kaligirwa N, Agaba S, Karema C, Mens PF, de Vries PJ. Pharmacovigilance of artemether-lumefantrine in pregnant women followed until delivery in Rwanda. *Malar J* 2012; **11**: 225.
8. Mosha D, Mazuguni F, Mrema S, Sevene E, Abdulla S, Genton B. Safety of artemether-lumefantrine exposure in first trimester of pregnancy: an observational cohort. *Malar J* 2014; **13**: 197.
9. Dellicour S, Desai M, Aol G, et al. Risks of miscarriage and inadvertent exposure to artemisinin derivatives in the first trimester of pregnancy: a prospective cohort study in western Kenya. *Malar J* 2015; **14**: 461.
10. Moore KA, Simpson JA, Wiladphaingern J, et al. Influence of the number and timing of malaria episodes during pregnancy on prematurity and small-for-gestational-age in an area of low transmission. *BMC Med* 2017; **15**(1): 117.
11. Moore KA, Simpson JA, Paw MK, et al. Safety of artemisinins in first trimester of prospectively followed pregnancies: an observational study. *Lancet Infect Dis* 2016; **16**(5): 576-83.
12. Saito M, Carrara VI, Gilder ME, et al. A randomized controlled trial of dihydroartemisinin-piperaquine, artesunate-mefloquine and extended artemether-lumefantrine treatments for malaria in pregnancy on the Thailand-Myanmar border. *BMC Med* 2021; **19**(1): 132.
13. Adam I, Elwasila E, Mohammed Ali DA, Elansari E, Elbashir MI. Artemether in the treatment of falciparum malaria during pregnancy in eastern Sudan. *Transactions of the Royal Society of Tropical Medicine and Hygiene* 2004; **98**(9): 509-13.
14. Adam I, Elhassan EM, Omer EM, Abdulla MA, Mahgoub HM, Adam GK. Safety of artemisinins during early pregnancy, assessed in 62 Sudanese women. *Annals of tropical medicine and parasitology* 2009; **103**(3): 205-10.
15. Ahmed R. Pregnancy outcomes in women exposed to dihydroartemisinin-piperaquine during first trimester of pregnancy in Indonesia- Final Study Report. Unpublished, 2019.
16. Deen JL, von Seidlein L, Pinder M, Walraven GE, Greenwood BM. The safety of the combination artesunate and pyrimethamine-sulfadoxine given during pregnancy. *Transactions of the Royal Society of Tropical Medicine and Hygiene* 2001; **95**(4): 424-8.
17. Dellicour S, Brasseur P, Thorn P, et al. Probabilistic record linkage for monitoring the safety of artemisinin-based combination therapy in the first trimester of pregnancy in Senegal. *Drug safety* 2013; **36**(7): 505-13.
18. Poespoprodjo JR, Fobia W, Kenangalem E, et al. Dihydroartemisinin-piperaquine treatment of multidrug resistant falciparum and vivax malaria in pregnancy. *PLoS One* 2014; **9**(1): e84976.
19. Rouamba T, Sondo P, Derra K, et al. Optimal Approach and Strategies to Strengthen Pharmacovigilance in Sub-Saharan Africa: A Cohort Study of Patients Treated with First-Line

Artemisinin-Based Combination Therapies in the Nanoro Health and Demographic Surveillance System, Burkina Faso. *Drug Des Devel Ther* 2020; **14**: 1507-21.
20. McGready R, Cho T, Keo NK, et al. Artemisinin antimalarials in pregnancy: a prospective treatment study of 539 episodes of multidrug-resistant Plasmodium falciparum. *Clin Infect Dis* 2001; **33**(12): 2009-16.
21. McGready R, Lee SJ, Wiladphaingern J, et al. Adverse effects of falciparum and vivax malaria and the safety of antimalarial treatment in early pregnancy: a population-based study. *Lancet Infect Dis* 2012; **12**(5): 388-96.
22. EUROCAT. EUROCAT Congenital anomalies prevalence charts and tables. https://eu-rd-platform.jrc.ec.europa.eu/eurocat/eurocat-data/prevalence_en (accessed 23 Feb 2022.
